# Combining Recurrence Analysis and Automatic Movement Extraction from Video Recordings to Study Behavioral Coupling in Face-to-Face Parent-Child Interactions

**DOI:** 10.3389/fpsyg.2017.02228

**Published:** 2017-12-19

**Authors:** David López Pérez, Giuseppe Leonardi, Alicja Niedźwiecka, Alicja Radkowska, Joanna Rączaszek-Leonardi, Przemysław Tomalski

**Affiliations:** ^1^Neurocognitive Development Lab, Faculty of Psychology, University of Warsaw, Warsaw, Poland; ^2^Faculty of Modern Languages and Literatures, Adam Mickiewicz University in Poznan, Poznan, Poland; ^3^Faculty of Psychology, University of Finance and Management, Warsaw, Poland; ^4^Faculty of Psychology, University of Warsaw, Warsaw, Poland

**Keywords:** parent-child interactions, infant, automatic movement extraction, recurrence quantification analysis (RQA), tracking-learning-detection (TLD)

## Abstract

The analysis of parent-child interactions is crucial for the understanding of early human development. Manual coding of interactions is a time-consuming task, which is a limitation in many projects. This becomes especially demanding if a frame-by-frame categorization of movement needs to be achieved. To overcome this, we present a computational approach for studying movement coupling in natural settings, which is a combination of a state-of-the-art automatic tracker, Tracking-Learning-Detection (TLD), and nonlinear time-series analysis, Cross-Recurrence Quantification Analysis (CRQA). We investigated the use of TLD to extract and automatically classify movement of each partner from 21 video recordings of interactions, where 5.5-month-old infants and mothers engaged in free play in laboratory settings. As a proof of concept, we focused on those face-to-face episodes, where the mother animated an object in front of the infant, in order to measure the coordination between the infants' head movement and the mothers' hand movement. We also tested the feasibility of using such movement data to study behavioral coupling between partners with CRQA. We demonstrate that movement can be extracted automatically from standard definition video recordings and used in subsequent CRQA to quantify the coupling between movement of the parent and the infant. Finally, we assess the quality of this coupling using an extension of CRQA called anisotropic CRQA and show asymmetric dynamics between the movement of the parent and the infant. When combined these methods allow automatic coding and classification of behaviors, which results in a more efficient manner of analyzing movements than manual coding.

## Introduction

The analysis of parent-child interactions (PCI) is crucial for the understanding of early human development (e.g., Sameroff and Fiese, 2000; Bronfenbrenner and Morris, 2006; Schaffer, 2009). In the course of PCI, both partners must coordinate their behaviors (such as vocalizations, eye gaze and movement). Even when considering the interactions with young infants the key characteristics of this coordination are timing (e.g., Jasnow et al., 1988; Hane et al., 2003) and synchrony in relation to each other's behaviors (e.g., Feldman, 2007). Optimally, caregivers adjust their behaviors (modality, timing, form and content) to the behaviors of the infant to meet their needs. Infants, in turn, are very sensitive to interpersonal contingencies (e.g., Murray and Trevarthen, 1985; Goldstein et al., 2003) and easily learn the causal relations between their own actions and consequences of these actions (Rochat and Striano, 1998). During the first year of life infants greatly improve their motor (e.g., Rochat, 1992; Rocha et al., 2012) and attention control (e.g., Johnson, 1990, 1994; Elsabbagh et al., 2013). They also learn to coordinate various social behaviors in different modalities (e.g., gaze and the expression of affect, Yale et al., 2003; Lavelli and Fogel, 2005). Finally, toward the end of the first year, infants begin to coordinate the focus of their attention in relation to the attention of their parents (Butterworth, 2006; Mundy and Newell, 2007). With the growing role of the infant in shaping the interactions during the first year of life, parents and infants in various everyday situations gradually improve their coordination (Rączaszek-Leonardi et al., 2013).

In developmental psychology the study of infant-parent interactions has relied predominantly on laborious, manual coding of individual behaviors, especially in microanalytic approaches to interactions (see e.g., Feldman, 2007). Coding interactions from video recordings is one of the most time-consuming and costly tasks in infancy research, taking up to 10 times the length of a video (Lasecki et al., 2014), which is a limitation in many projects. Additionally, finding a finite set of well-defined categories of behaviors and attaining high interrater reliability is often very difficult since new research questions require some modifications in the coding scheme. Likewise, some adjustments need to be made to the coding scheme when dealing with different age groups. In many cases introducing changes into a coding scheme requires re-analyzing the same videos all over again. For these reasons applying various methods of automatic movement extraction and classification in already recorded videos might help extract additional information and to pick up the most important aspects of behavior for each study saving considerable amounts of time.

Quantification of movement in infancy research has been carried out by placing various invasive instruments, such as sensors or head cameras, on both the parent and the infant, often in combination with complex and expensive camera settings (e.g., Pereira et al., 2009; Karch et al., 2010). Compared to that effort, there were relatively few attempts to quantify infant movement in pre-recorded videos (Rahmati et al., 2014; Mirsharif et al., 2016). This kind of approach increases the ecological validity of research in human interactions and considerably reduces the costs and burden of having to rely on sophisticated movement-tracking technology. For instance, Rahmati et al. (2014) successfully applied motion segmentation techniques to obtain movement data from video recordings in infants at risk of developing cerebral palsy. However, these motion segmentation techniques do not perform as well in the case of complex images, where there is no prior knowledge of the expected movement of each object due to reduced quality of flow fields (i.e., apparent motion of brightness patterns in a visual scene caused by the relative motion between an observer and a scene, Rahmati et al., 2015) and they would likely cause tracking errors in PCI.

More generally, automatic quantification of movement in dyadic human interactions has been applied to study e.g., nonverbal courtship communication (Grammer et al., 1999) or interactional synchrony during conversations (e.g., Ramseyer and Tschacher, 2011; Paxton and Dale, 2013). But the automatic techniques employed in these studies (e.g., Motion Energy Analysis, Ramseyer and Tschacher, 2011) are sensitive to luminance changes. In general, they require stable camera conditions and people in the image should not occlude each other. The aforementioned studies showed that such problems could be minimized in adult studies, where instructions can be provided to and followed by the participants. However, infant studies are more challenging since infants do not follow instructions, the parents often occlude the infant in the field of view of the camera or the researchers need to manipulate the position of a remote-controlled camera in order to improve the view.

Recent approaches have sought to overcome these problems and employ automatic extraction of coordinated interaction from facial expressions during PCI (Messinger et al., 2009, 2010). Messinger et al. (2009, 2010) modeled infant and mother facial movements using Automated Facial Image Analysis. They used active appearance models (AAMs) which are algorithms for matching a statistical model of object shape and appearance to a new image. Subsequently they applied windowed cross-correlation analysis and showed that ongoing smiling activity of one partner predicted subsequent smiling activity of the other partner. However, AAMs need to be trained using a set of images, together with coordinates of landmarks that appears in those images. Moreover, AAMs are typically developed to solve a specific problem (e.g., to track face features) and the ability of the model to solve a new problem (e.g., to track hand movements) depends on the versatility of the model itself. Therefore, in the current study, we outline a different approach in which we automatize the coding process using a state-of-the-art tracking algorithm called Tracking-Learning-Detection (TLD, Kalal et al., 2010) to obtain fine grained and reliable movement quantification from videos of interactions of parents and 5.5-month-old infants. In contrast to AAMs, TLD does not need initial training and the algorithm is capable of tracking an object, by learning its shape despite changes in luminosity and other visual parameters and detecting it frame-by-frame both in real-time and in pre-recorded videos. Initially, we studied the applicability of TLD to extract movement from pre-recorded PCI in different situations. Our analysis is focused on PCI episodes, where the parent and the infant spontaneously oriented face to face while the parent was animating an object.

Moreover, we applied TLD for the first time in PCI videos in combination with Cross-Recurrence Quantification Analysis (CRQA, Coco and Dale, 2014) in order to investigate the coupling between movement of the parent and the infant in parent-child interactions. CRQA is emerging as one of the most useful methods when exploring the coupling of two behavioral signals from different actors engaged in a social interaction (e.g., Shockley et al., 2003; Richardson and Dale, 2005; Shockley and Turvey, 2005; Stephen et al., 2009). CRQA in comparison to linear methods such as cross-correlation, provides a more effective tool to determine whether, and to what extent, two systems produce common dynamics (Shockley et al., 2003). Thus, we conducted this nonlinear dynamical analysis on time series extracted with TLD to test whether individual movements of each partner are coupled in time. This approach additionally allows to test whether leader-follower relationships are in place, i.e., to test which one of the partners is the one mostly driving such coordinative pattern. Finally, we applied anisotropic Cross-Recurrence Quantification Analysis (aCRQA, Cox et al., 2016) to assess the quality of this coupling between both partners in the interaction. To illustrate this approach, we provide a step by step example of our methodology (see Supplementary Tutorial).

## Materials and methods

### Participants

Data presented in this paper were collected as part of a larger longitudinal study from July 2013 through August 2016. The mean infant age in the sample was 156.57 days (*SD* = 14.20, range 134–179 days) with 8 boys and 13 girls. All infants were healthy and born full-term. Participants were Caucasian, predominantly middle-class families living in a city with >1.5 million inhabitants. The mother was indicated as the primary caregiver for all infants and none of the infants attended a nursery. Additionally, a 9-month-old girl took part in a separate study to demonstrate the suitability of the method to extract and analyse movement in various situations (see section Procedure on p. 7–8).

Participants were recruited through flyers and posters in local healthcare facilities, nurseries and through media ads. All parents gave written informed consent prior to the testing. The study was approved by the local institution's ethics committee and conformed to the Declaration of Helsinki.

## Equipment

All interactions were recorded in a laboratory room, on a carpeted play area, with a uniform set of age-appropriate toys using 3 remote-controlled CCTV IP color cameras in SD quality (752 × 582 pixels). The first camera was placed low on the wall to capture the infant's' visual behavior, the second camera was placed higher relative to the first camera, in the opposite corner of the play area. The third camera was placed near the ceiling, in the third corner of the play area and captured the whole room. The image from cameras was recorded via an Ethernet connection with a sampling frequency of 25 frames per second.

The additional interaction (see page 7–8) was recorded with a remote-controlled CCTV color camera in SD quality (752 × 582 pixels) and a Sony camera Model PJ650VE Full HD (1,080p). The latter camera was located perpendicular to the interaction.

## Procedure

In this study, we selected videos of 21 infant-parent dyads from a free-play task collected as part of a larger research project (from a total of 119 available recordings; see Niedzwiecka et al., 2017 for the description of the project protocol). During the interactions parents were instructed to play with their infants in the same way they did at home. We selected predefined episodes, each coming from a different participant and lasting at least 15 s. For each episode we chose the recording from the camera that provided the best field of view of the entire interaction. In these episodes, the parent and the infant were oriented face to face while the parent was animating an object in front of the infant during the whole duration of the episode. The average length of these episodes was 23.51 s (*SD* = 8.36 s, range 15–41.08 s). The remaining 98 recordings were excluded from the analysis because an episode of face-to-face interaction was not observed or because such episodes lasted <15 s.

In addition, we separately recorded structured episodes of infant-parent interactions of another dyad in order to demonstrate that movement dynamics can be extracted from different tasks. Two independent scenarios were examined. The first one involved playing with a spinning toy for 3 min while the second one consisted of a spoon-feeding task of the same duration. In both tasks the mother and the infant were facing each other, with the infant sitting in a baby seat and the mother sitting on the floor opposite the infant.

### Data analysis

#### Tracking-learning-detection

TLD is a recently developed framework that allows tracking objects in real-time or pre-recorded video streams (Kalal et al., 2010). The objects do not need to be unequivocally defined in all their features in advance, but rather simply selected at the beginning of the video in a rectangular bounding box defining the object of interest. The algorithm learns the pattern enclosed in the selected area and tracks this object by estimating its motion across frames under the assumption that the movement is limited and the object is visible. Additionally, it detects the object within the image and makes use of different appearances observed and learned in the course of the video. Finally, a learning process continuously checks the performance of both the tracker and the detector in order to correct errors (i.e., missed and false detections). This learning process helps the detector to generalize to more object appearances and to discriminate the object from the background (Kalal et al., 2010).

**Figure 1:**
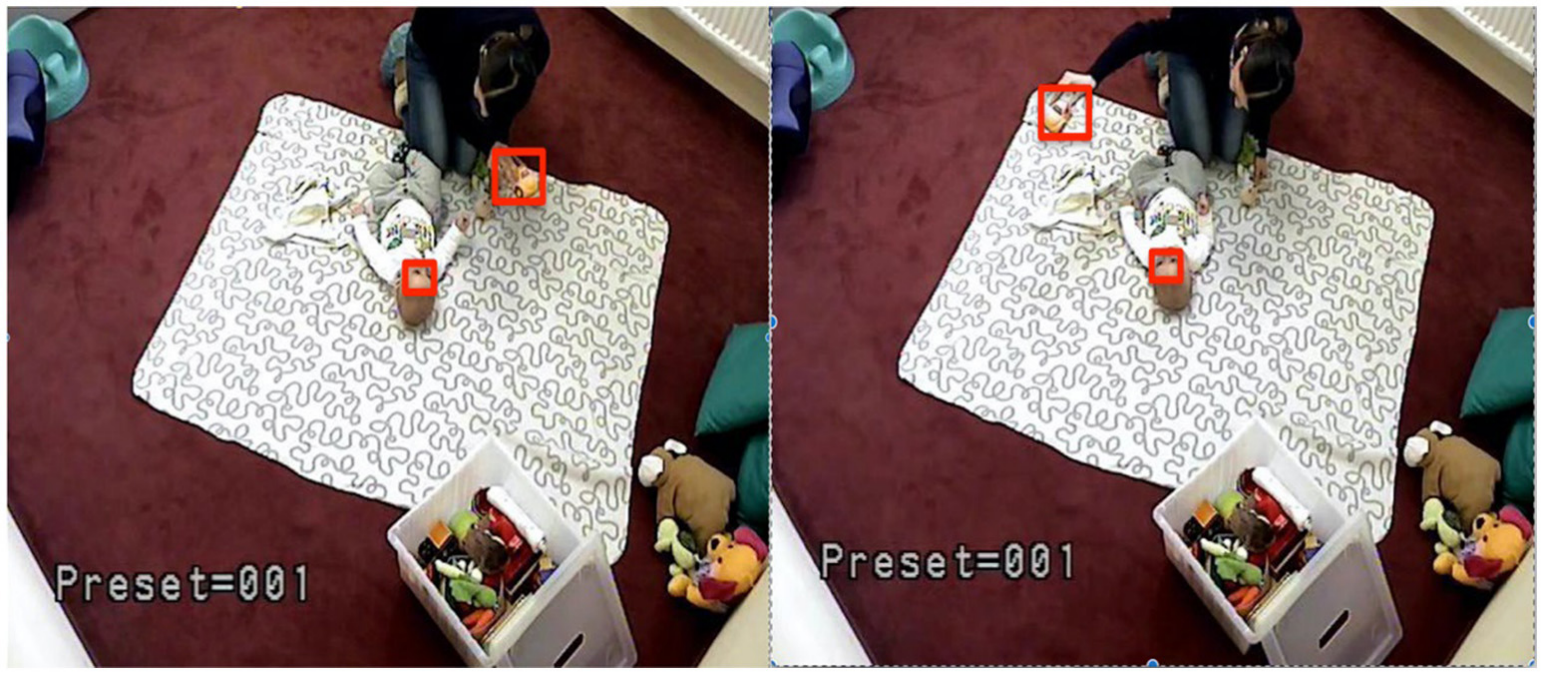
Example of how TLD was used to track the infant's focus of attention and the object that the mother held in front of the infant. The red bounding boxes represent these tracked features. Parents gave written consent to use the images in the publication.

**Figure 2:**
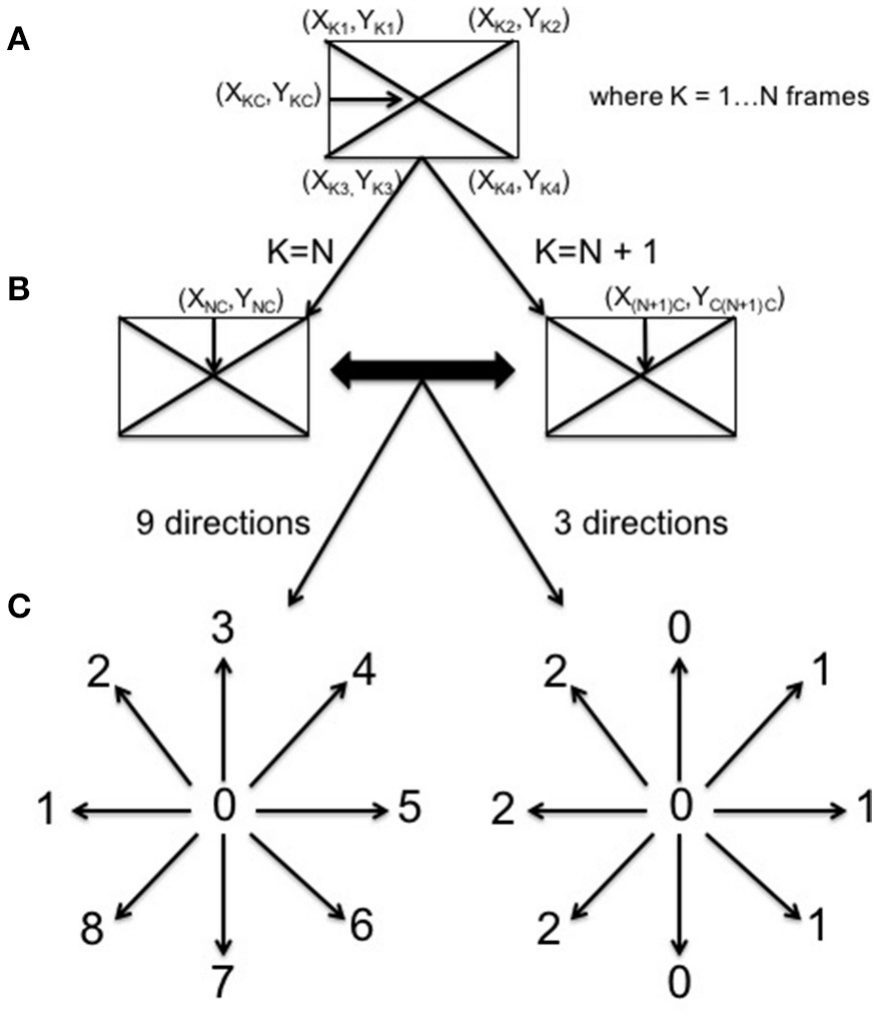
Categorization of movement. The central coordinates of the TLD window are extracted **(A)** and compared to the next window central coordinates **(B)**. The movement was classified following two systems of coordinates containing 9 and 3 categories of movement directions, respectively **(C)**.

In this study, TLD was used to track, on one side, the infants' focus of attention by following the movement of his/her face and, on the other, the object which the mother holds in front of the infant during the interaction (see Figure 1). TLD returned a set of coordinates for each tracked feature. In order to analyse this data in CRQA each set of coordinates was subsequently translated into a categorical time series indicating the direction of movement (e.g., left, up-left, down-left). Figure 2 illustrates this process. First, in every frame, each set of coordinates was translated into the central coordinates of the tracked feature (Figure 2A). Then, these coordinates were compared with the central coordinates of the next frame to determine the direction of movement (Figure 2B). Finally, this movement was classified following two tentative systems of coordinates (Figure 2C). The first one contained 3 categories (simple coordinate system): 0 for no movement in right or left direction, 1 for right and 2 for left. The second one had nine different categories (detailed coordinate system): 0 for no movement, 1 for left direction, 2 for up-left, 3 for up, 4 for up-right, 5 for right, 6 for down-right, 7 for down, and 8 for down-left.

The performance of the tracker was visually inspected to avoid false positives. However, in some cases, the tracker was unable to follow for some frames the specific object or the position of the infant's focus of attention. Due to the specificity of the analyzed episodes, where the parent normally animated the object from left to right and *vice versa*, a linear movement was assumed in those missing frames. Therefore, a linear interpolation was calculated between the last known point and the first one available (see section Results on p. 15). The accuracy of the tracker was computed calculating the percentage of successfully tracked data points from the total number of frames in the video (e.g., prior to the linear interpolation process).

### Cross recurrence quantification analysis

We used CRQA to quantify the coordination of movements between infants' head movements and objects animated by parents. CRQA is an extension of Recurrence Quantification Analysis that compares two different time series and extracts the pattern of matching states at all lags (Zbilut et al., 1998). We aligned the two categorical movement direction time series extracted from TLD and applied diagonal-wise CRQA (see e.g., Dale et al., 2011b) to measure their temporal coupling. Positive matches between time series are represented with a point in the recurrence plot, which represents the global structure of recurrence (Dale et al., 2011a). The analysis of recurrence rate near the main diagonal line of the recurrence plot allows to reconstruct a lag profile, which contains information about the coordination of those time series (Richardson and Dale, 2005; Leonardi et al., 2016). Each diagonal on the recurrence plot corresponds to a particular delay or lag in the alignment between the parent's and the infant's movements. For each episode, a lag profile between −4 and +4 s was calculated in Matlab (MATLAB 2016a, The MathWorks, Inc., Natick, Massachusetts, United States) using a translated version of the *drpdfromts* function present in the CRQA R-package (Coco and Dale, 2014). The lag profile does also provide additional information about the leading actor during the interaction (e.g., Shockley et al., 2003; Dale et al., 2011a; Leonardi et al., 2016). In our case, negative values of the profile indicate a parent-leading role while positive values an infant-leading one.

The distribution profiles were compared to two different baseline conditions (Richardson and Dale, 2005). This step is necessary to validate that the patterns within the diagonal profiles obtained from the recurrence plots are real and do not arise by chance or due to situational factors. The first baseline was calculated using the real data from parent movement and a shuffled version of the infant movement. In this case, the temporal order of the infant time series was shuffled and its recurrence computed with the original parent time series. By shuffling the infant time series, the individual recurrences are distributed evenly over the time series and this baseline represents the likelihood that the profile is generated accidentally or by one movement randomly following the other (Richardson and Dale, 2005; Dale et al., 2011a). The second baseline was obtained by random-pairing. Here, the recurrence was averaged between the parent and five other randomly selected infants. This baseline controls that the profile is not task-specific. Task-specific recurrence refers to the baseline of recurrence between infants who share the same free-play task but show different behavior (Richardson and Dale, 2005). During random-pairing the time series may possess different length. In this case, the last part of the longest time series was trimmed to match the length of the shortest one.

### Statistical analysis

To assess any statistical difference among the recurrence profiles obtained and the baseline profiles taken as control conditions in this comparison, we used linear mixed effects modeling. The chosen model was similar both in the case of the detailed coordinate system and in the case of simple coordinate system. In the statistical model fitted we used two variables as fixed effects (Condition and Lag) and one (Dyad) as the random effect. The dependent variable modeled was the level of Recurrence Rate in the profile. Both in the case of shuffled baseline condition and in the case of false pair baseline condition we used the fixed effect Condition and compared its two levels, i.e., baseline vs. experimental, on the outcome variable Recurrence Rate at the intercept. In the case of the second fixed effect (Lag), we used a subset of the lags in the original profiles (corresponding to a coarser grained time sampling at 6.25 Hz from −4 to +4 s) reducing hence the number of lags (i.e., the levels of this variable) entered into the analysis from 201 to 51. This considerably reduced the number of coefficients to be estimated in the analysis (from 404 to 104). The second fixed effect (Lag) compared the value of Recurrence Rate at each of the 51 lags with the reference level taken at lag −4 s. The full model fitted the fixed effects singularly and in interaction between them, in which case the value of Recurrence Rate for the level of experimental Condition at each Lag was contrasted with the reference value (i.e., baseline Condition at Lag −4 s). We also built and fitted two additional and more general models in order to proceed with model comparison through likelihood-ratio tests. One of these models did not take into account the interaction of Condition and Lag, while the second was the minimal “null” model, with no fixed effects and only the random effect. The analyses were run in R (R Core Team, 2016) using packages lme4 (Bates et al., 2015) and lmerTest (Kuznetsova et al., 2016), which allow to check the statistical significance for the estimated coefficients of the model using *t*-tests and the Satterthwaite approximations for the degrees of freedom.

Bonferroni-corrected *t*-tests were applied to compare the individual profiles during the spinning and the feeding tasks. Thus, each profile was divided into four windows of equal size and individual *t*-tests were performed for each one. *Post-hoc* Bonferroni correction was applied by dividing the significant test alpha (0.05) by the number of windows. Only those *t*-tests whose *p*-values were lower than the adjusted alpha level were considered significant.

### Anisotropic CRQA

In CRQA, the nature of categorical time series impacts the recurrence plots in such way that most of the recurrences are arranged to form rectangular or vertical line structures. However, most of the traditional RQA measures focused on quantifying diagonal structures (Webber and Zbilut, 1994). Thus, recently, a new technique, called Anisotropic CRQA (aCRQA), has been proposed to overcome this problem (Cox et al., 2016). Instead of analyzing the diagonal structures, aCRQA can provide information about the coupling between two time series by quantifying the vertical and horizontal structures in the plot. Three measures are considered in this case (Cox et al., 2016):

- Laminarity (*Lam*): is the proportion of recurrences that are part of the vertical or horizontal lines, and indexes the general level of persistence in some particular state of one of the time series.
- Trapping Time (*TT*): is the average length of either the vertical or horizontal lines. In the recurrence plot, it indexes the average time spent by the participants in the various movement categories mapped in the time series. It was calculated here using the *tt* function from the CRP Toolbox for Matlab (Marwan et al., 2002).
- Maximum Line (*MaxL*): represents the length of the longest vertical or horizontal line and hence gives the longest time spent in a single state by interaction partners.

It has been recently proposed that the directionality of the asymmetry between vertical and horizontal lines in a recurrence plot can provide complementary information about the coupling of two subsystems (Cox et al., 2016). De Jonge-Hoekstra et al. (2016) applied cross-recurrence to analyse gestures and speech data streams recorded in children during a hands-on science task and found that in 5-year-old children such asymmetry in the resulting recurrence plots suggests that speech categories attract gesture categories at the same level of abstraction in a more dynamically stable fashion than *vice versa*. They also showed that gestures and speech are more synchronized when children grow older. In our case, any asymmetry found between vertical and horizontal lines would reveal an asymmetric dynamic attunement between the movement of the parent and the infant. For example, if the movements of the mother and infant recur and have the same duration in time a perfect symmetry would be found. However, such coordination during interactions is difficult to achieve and the asymmetry between movements starts to arise. Here, we expect an asymmetric dynamic attunement between the movement of the parent and the infant since the movement of the parent is likely more continuous and lasts longer in comparison with the movement of the infant.

## Results

### Moving toy

TLD was able to track the object and the infant's direction of looking with an overall average accuracy of 94.65% (*SD* = 10.11), leaving only a small portion of missing values. Separately, the infant's direction of looking was tracked more efficiently with an average accuracy of 98.48% (*SD* = 5.15) in comparison to the object 90.81% (*SD* = 12.34). Particularly, the tracker was able to follow the infant's direction of looking with an accuracy of 100% in 18 out 21 cases. Likewise, the tracker kept up with the object with accuracy >90% in 15 out of 21 cases, with only one case below 75% accuracy because the object was moved too fast during the interaction. All missing values were linearly interpolated between the last known point and the first one available. The number of interpolated points varied from 0 to 304 for the object and from 0 to 86 for the infant's direction of looking (total points ranged from 375 to 1,027). The average number of interpolated points was 6.62 (*SD* = 20.03) for the infant's direction of looking and 47.81 (*SD* = 71.92) for the object.

**Figure 3:**
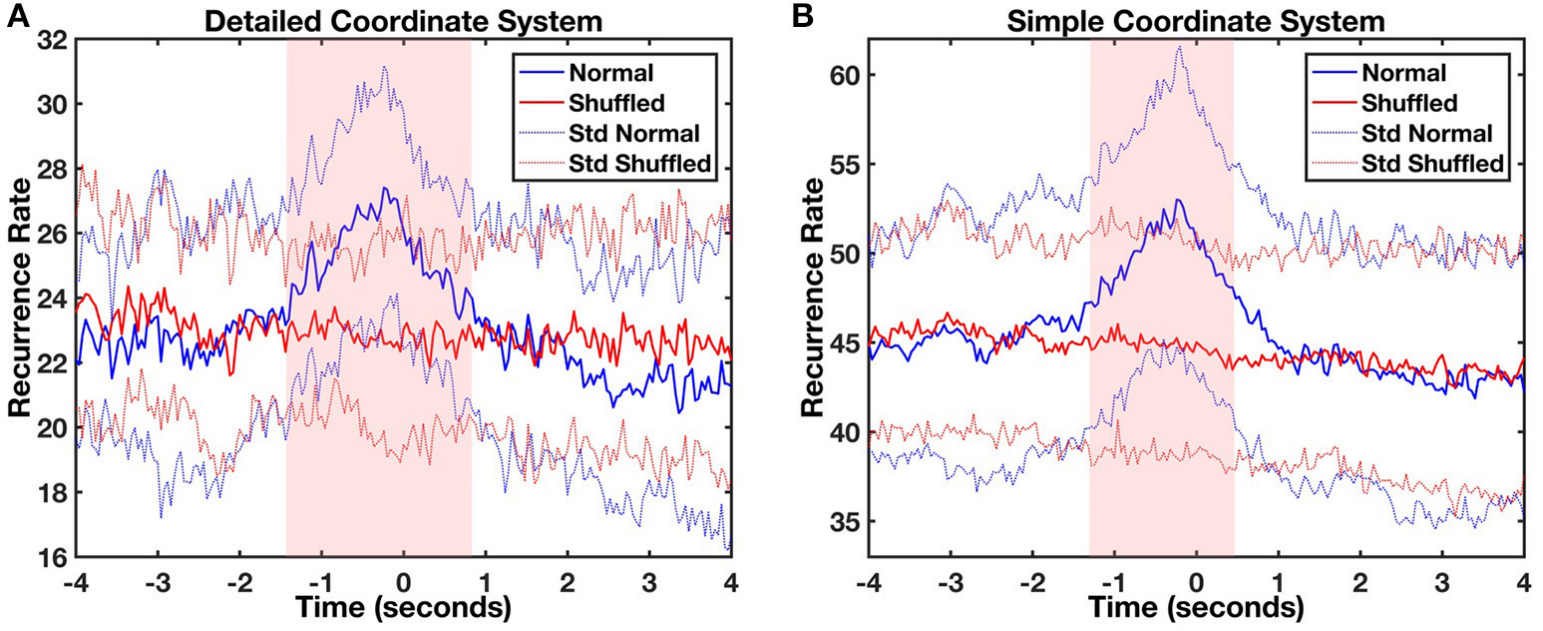
Average object-infant's focus of attention (blue) and object-shuffled infant (red) lag profiles computed using diagonal-wise CRQA for the detailed **(A)** and the simple **(B)** coordinate systems. The light red and light blue lines represent the standard deviation of mean normal and shuffled profiles, respectively. The red shaded area represents the time window where significant differences were found between the original (blue) and control shuffled profiles (red).

Lag profiles of recurrence were computed using diagonal-wise CRQA for both types of movement categorization (Figures 3A,B). The maximum recurrence of the average profile between the infant and parent movements was observed in both cases at a lag of −240 ms. In other words, the parent moved the object in front of the baby and after 240 ms the infant followed it with head movement. The statistical comparison of CRQA lag profiles with randomized baseline profiles of the same time series showed that this coupling was not produced accidentally or by random head movement. A likelihood ratio test compared the full linear mixed model (both additive and interaction effects) against more general null-models (additive only or no effects). The test showed that the full model is highly significant for both analyses compared to the additive only model [detailed coordinate system: $\chi_{\left(50\right)}^{2}$ = 234.54, *p* < 0.001, *V* = 0.52; simple coordinate system: $\chi_{\left(50\right)}^{2}$ = 287.39; *p* < 0.001, *V* = 0.47], which in turn is also highly significant compared to the null model [detailed coordinate system: $\chi_{\left(51\right)}^{2}$ = 220.14, *p* < 0.001, *V* = 0.45; simple coordinate system: $\chi_{\left(51\right)}^{2}$ = 6832.17, *p* < 0.001, *V* = 2.55]. When looking at the significance of the modeled coefficients of the full model, in the analysis with the simple coordinate system significant coefficients of the interaction between Lag and Condition were found at lags between −1.28 and 0.48 s (*t*s > 2.074, *p*s < 0.05, *d*s > 0.046), while in the detailed coordinate system they ranged between lag −1.44 and lag 0.8 s (*t*s > 1.991, *p*s < 0.05, *d*s > 0.046). This confirms that the recurrence at these lags arises from a real process of coordination of the movements of mothers and infants, and is significantly different from the shuffled baseline level.

**Figure 4:**
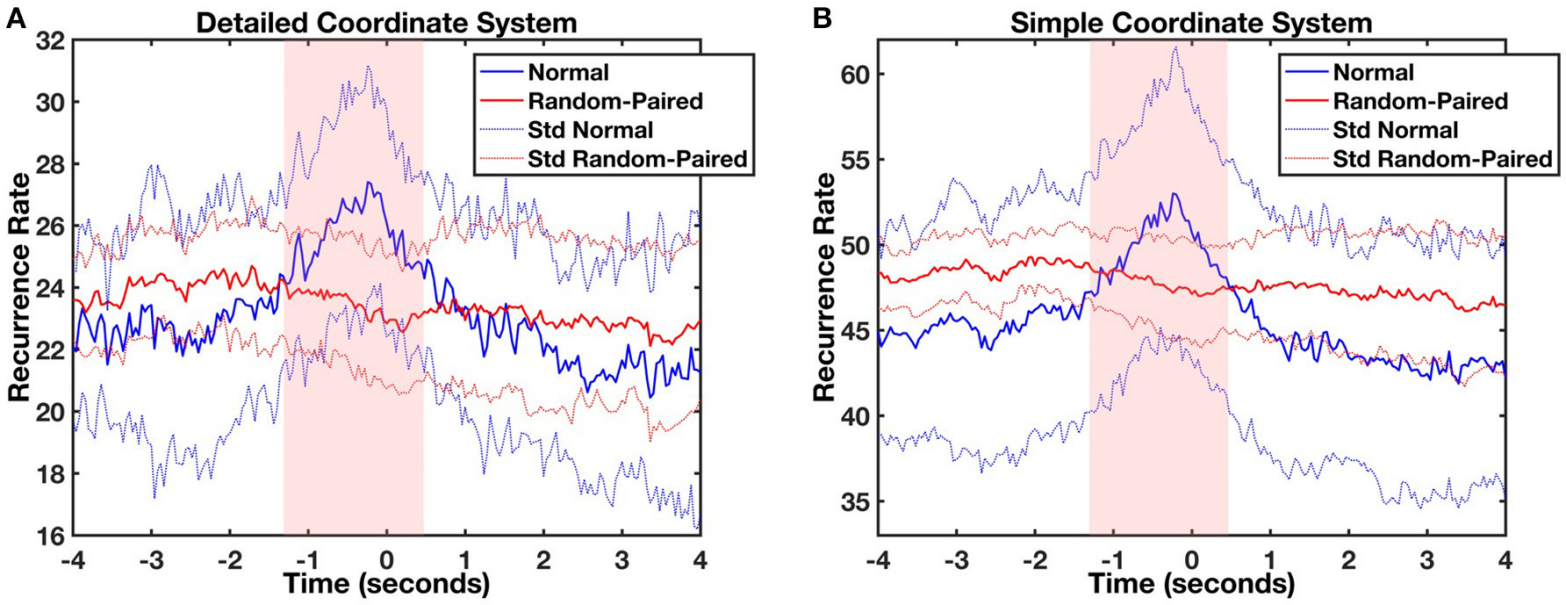
Average object-infant's focus of attention (blue) and object-randomized infant's focus of attention (red) lag profiles computed using diagonal-wise CRQA for the detailed **(A)** and the simple **(B)** coordinate systems. The light red and light blue lines represent the standard deviation of mean normal and random-paired profiles respectively. The red shaded area represents those times where significant differences were found between both profiles.

Next, we tested whether the coupling was due to infants sharing the same free-play task by randomly pairing each mother with 5 different infants (Figure 4). No coupling was found between the mother's movements and the movements of any of the randomly-paired infants. A likelihood ratio test showed again that the full linear mixed model was highly significant in comparison with the additive model for both the analyses [detailed coordinate system: $\chi_{\left(50\right)}^{2}$ = 288.49, *p* < 0.001, *V* = 0.52; simple coordinate system: $\chi_{\left(50\right)}^{2}$ = 287.89; *p* < 0.001, *V* = 0.52], and the additive model was significantly different from the base (null) model [detailed coordinate system: $\chi_{\left(51\right)}^{2}$ = 6872.94, *p* < 0.001, *V* = 2.55; simple coordinate system: $\chi_{\left(51\right)}^{2}$ = 6827.09, *p* < 0.001, *V* = 2.55]. Even in this case the significant coefficients of the interaction between Lag and Condition were located in a very specific range around lag 0, comparable to the one already registered in the previous analyses. In both cases (for the simple and the detailed coordinate systems) this interval ranged from Lag −1.28 and 0.48 s (*t*s > 2.07, *p*s < 0.05, *d*s > 0.046). This shows that the effect of movement coupling in mother-infant dyads as captured by cross-recurrence profiles is significantly different than the random-paired baseline level and it cannot be attributed to the task itself. Some task-related recurrence is, however, present since the random-paired baseline has higher overall recurrence in comparison to the shuffling baseline. This is more visible in the simple coordinate system where the mean recurrence rate (RR) shuffled and RR random-paired were respectively equal to 43.99 (*SD* = 2.05) and 47.52 (*SD* = 0.61) in comparison with the detailed coordinate system where the mean RR shuffled and the mean RR random-paired were 22.89 (*SD* = 0.51) and 23.41 (*SD* = 0.59).

**Figure 5:**
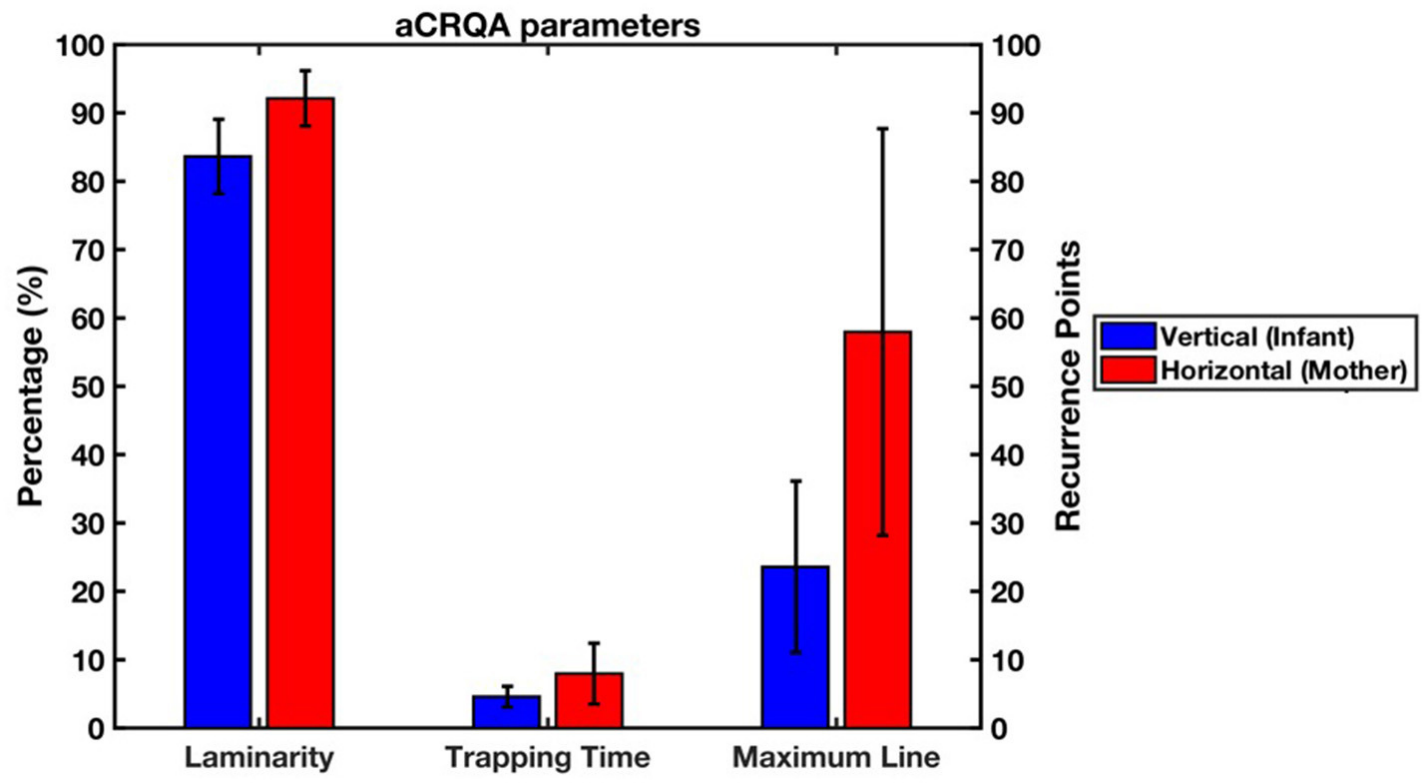
Means and standard deviations of the laminarity, trapping time and maximum line from the aCRQA analysis (blue represents vertical lines and red horizontal ones). Laminarity is measured in percentage (%) while trapping time and maximum line are measured in recurrence points. The mother's movement was represented along the horizontal axis, while the infant‘s movement along the vertical axis of the recurrence plot.

To investigate the asymmetry in the coupling of the two subsystems, or in other words, the directionality of the asymmetry between vertical and horizontal lines in the recurrence plot, we applied aCRQA. We looked at the proportion of recurrences that are part of the vertical or horizontal lines (i.e., laminarity, Lam), the average length of these vertical or horizontal lines (i.e., trapping time, TT) and the longest vertical or horizontal line (i.e., maximum line, MaxL). Results from the aCRQA analysis on the vertical (i.e., subscript v) and horizontal (i.e., subscript h) lines are presented in Figure 5. Lam_V_ ranged from 0.78 to 0.97, meaning that 78.37–97.27% of the recurrent points form vertical lines. TT_V_ varied from 2.98 to 8.26 average recurrence points, or in other words, the infant movement follows the mother movement with an average duration from 74.50 to 206.5 ms. MaxL_V_ ranged from 7 to 52 recurrence points, showing that the maximum time the infant is trapped within the mother movements lasted from 175 to 1300 ms. Horizontal values were higher in comparison to the vertical ones with Lam_H_ fluctuating from 0.83 to 0.99, TT_H_ from 2.44 to 18.59 and MaxL_H_ from 4 to 134.

Paired-samples *t*-tests showed that at a group level for all three measures the averages for the horizontal lines were significantly higher than for the vertical lines [LAM, *t*_(20)_ = −5.59, *p* < 0.01, *d* = −1.77; TT, *t*_(20)_ = −3.30, *p* < 0.01, *d* = 1.008 and MaxL, *t*_(20)_ = −5.31, *p* < 0.01, *d* = −1.50]. This reveals an asymmetry in the dynamics between the parent and infant movements. It suggests that the infants' movement in one direction is more irregular compared to the parents' movement in one direction, which was more continuous and regular.

### Feeding task

**Figure 6:**
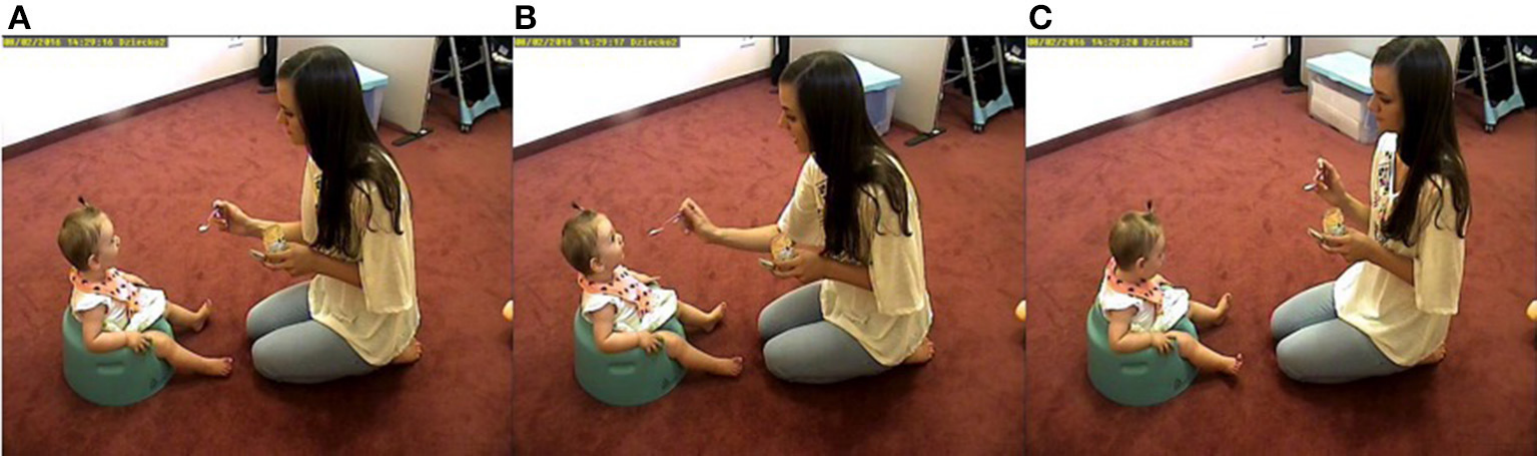
Example frames of the interaction during the feeding task. First, the mother leans forward to feed her baby **(A)**. Then, the baby leans back anticipating the food while looking at her mother **(B)**. Finally, the mother and the baby move back to the original position **(C)**. This process is repeated throughout the interaction. Parents gave written consent to use the images in the publication.

TLD was able to track whole-body movements of the infant and the mother with an overall accuracy of 100% in both cases since the tracker did not report missing values. Example frames from the recordings of the interaction can be seen in Figure 6. During the interaction, the mother repeatedly moves back and forth feeding the infant, who also moves accordingly. Given that we tracked whole body movements and that in the previous task (moving toy) the simple coordinate system captured the movement adequately, we decided that a more fine-grained categorization was not necessary since the baby seat restricts the infant movement (see Supplementary Materials for the results for the detailed coordinate system).

**Figure 7:**
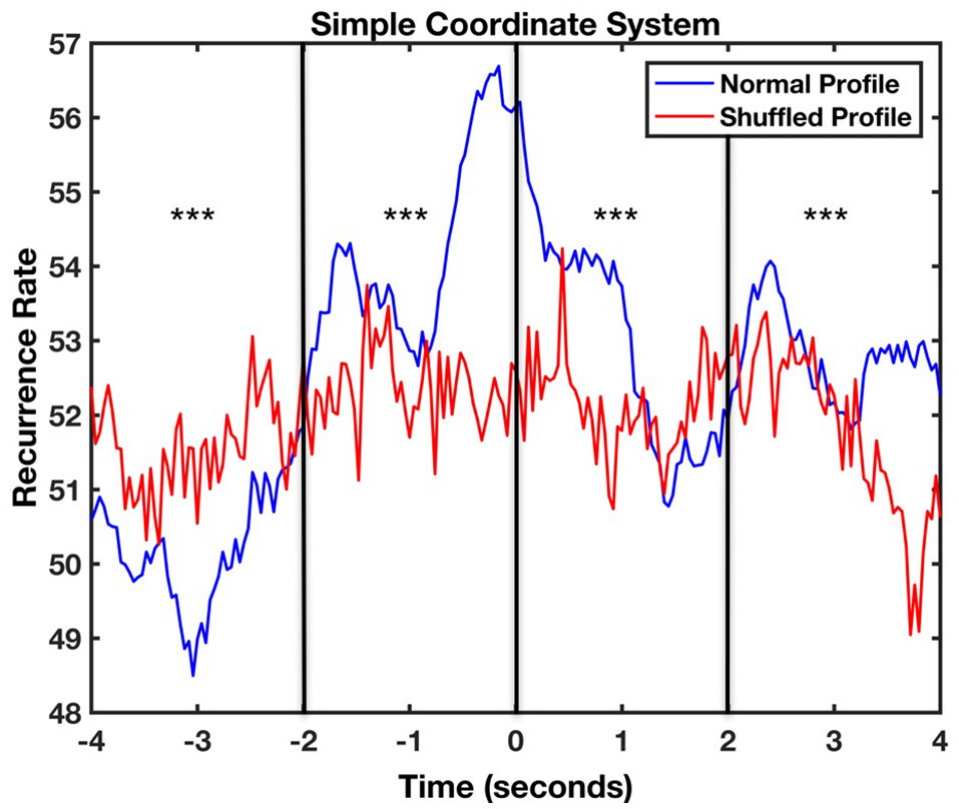
Lag profile between mother and infant body movements during feeding task computed using diagonal-wise CRQA for the simple coordinate system. The asterisks represent in which windows there were significant differences between the original recurrence profile with the shuffled one (^***^p < 0.001).

Following the same procedure as previously, we carried out a CRQA analysis to calculate the lag profiles of recurrence. Figure 7 represents the lag profiles computed for the simple coordinate system. The maximum recurrence between movements of the infant and the mother was observed at a lag of −200 ms. This means that the mother initiated an action that was followed by the infant after approximately 200 ms. Bonferroni-corrected *t*-tests showed significant differences between the original recurrence profile with the shuffled one in the four windows [Time Window 1: *t*_(49)_ = −13.89, *p* < 0.001, *d* = −2.75; Time Window 2: *t*_(49)_ = 5.23, *p* < 0.001, *d* = 2.01; Time Window 3: *t*_(49)_ = 9.16, *p* < 0.001, *d* = 1.17; Time Window 4: *t*_(49)_ = 6.54, *p* < 0.001, *d* = 1.33]. This suggests that the movements of mother and infant are coupled also in this task and are not produced by chance.

Finally, aCRQA was applied to study the asymmetry between vertical and horizontal lines in the recurrence plots. Visual inspection revealed lower asymmetry in this case (Lam_V_, = 0.91, TT_V_ = 9.77, MaxL_V_ = 71, Lam_H_, = 0.96, TT_H_ = 9.63, MaxL_V_ = 136) suggesting increased coordination in the movements of the parent and the infant during this task.

### Spinning toy task

**Figure 8:**
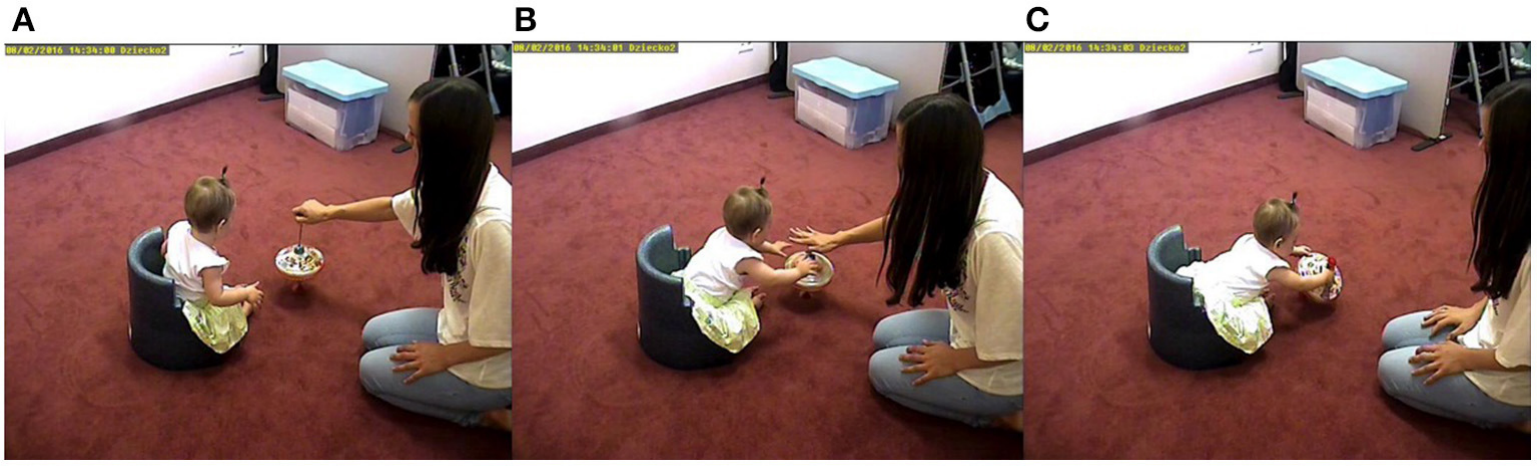
Example frames of the interaction during the spinning toy task. First, the mother leans forward and shows the functioning of the spinning toy to the infant **(A)**. The infant interested leans forward to play with the object **(B)** while the mother leans back **(C)**. This process is repeated throughout the interaction. Parents gave written consent to use the images in the publication.

Once again, TLD was able to track the infant and mother whole body movements with an overall accuracy of 100% for both partners. Example frames from the recordings of the interaction during the spinning toy task can be seen in Figure 8. During the interaction both the parent and the infant recurrently move back and forth interacting with the spinning toy. In the same way as in the feeding task, here we tracked whole body movements and used a simple coordinate system to capture the movement (see Supplementary Materials for the results of the detailed coordinate system).

**Figure 9:**
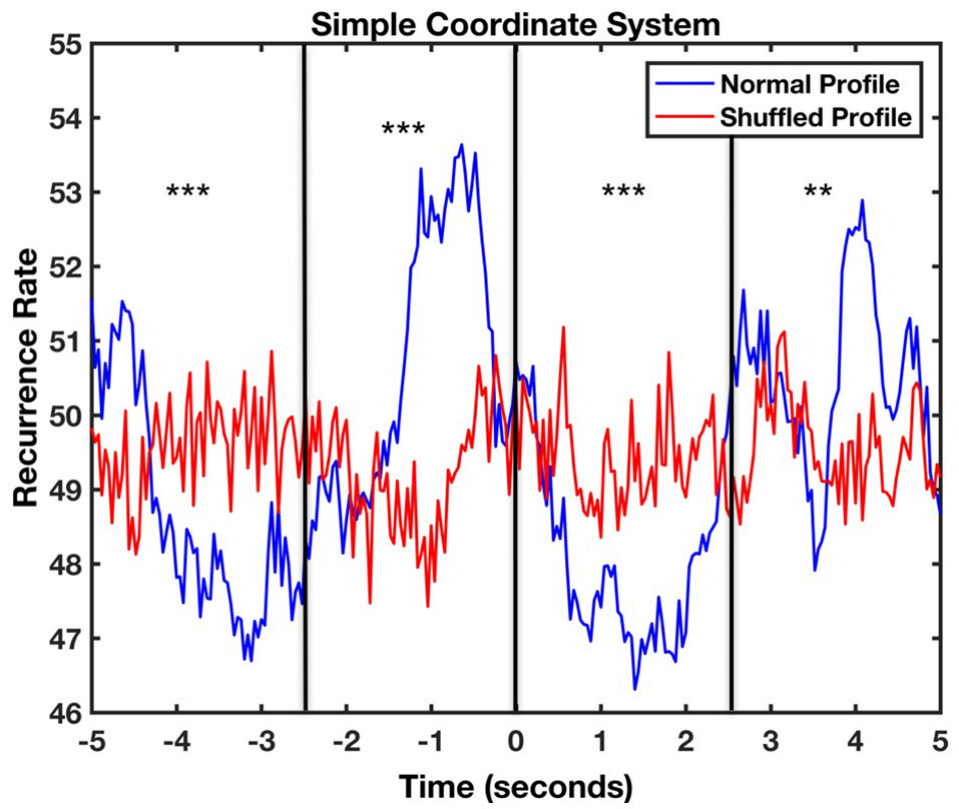
Lag profile between mother and infant body movements during the spinning toy task computed using diagonal-wise CRQA for the simple cosordinate system. The asterisks represent in which windows there were significant differences between the original recurrence profile with the shuffled one (^**^p < 0.01, ^***^p < 0.001).

Since the dynamics of movement in the spinning toy task are more complex, we extracted the profiles from −5 to +5 s in case later peaks arise (Figure 9). Once more, we carried out a CRQA analysis to calculate the lag profiles of recurrence. The maximum coordination between categorized movements when using the spinning toy was found at −640 ms. Interestingly, the plot shows two minimum values located at +1400 and −3120 ms suggesting low recurrence between movement categories at those times. The comparison with the randomized baseline profile of the infant movement visually shows again that this profile was not produced accidentally or by random playing with the object. Bonferroni-corrected *t*-tests showed significant differences between the original recurrence profile with the shuffled one in the four windows [Time Window 1: *t*_(61)_ = −5.96, *p* < 0.001, *d* = −0.07; Time Window 2: *t*_(61)_ = 5.16, *p* < 0.001, *d* = 0.85; Time Window 3: *t*_(61)_ = −9.67, *p* < 0.001, *d* = −2.43; Time Window 4: *t*_(61)_ = 3.16, *p* = 0.002, *d* = 0.70]. The positive sign of the *t*-test in the second window in comparison to the first and the third window suggests that the coordinative recurrence peak concentrates in that area while in other windows there is actually less recurrence than the random level. This shows that the movements of mother and infant are coupled also in this task and again are not produced by chance.

Finally, aCRQA was applied and asymmetry was again observed in this case (Lam_V_, = 0.83, TT_V_ = 5.35, MaxL_V_ = 54, Lam_H_, = 0.93, TT_H_ = 8.44, MaxL_H_ = 76) where the mother movement is more regularly displaying the same direction of movement compared to the infant. Following the results of the moving toy task this suggest that the infant's movement in one direction is more irregular compared to the mother's movement.

## Discussion

Our goal was to test the feasibility of conducting automated movement analysis in pre-recorded videos of parent-child interactions (PCI) as a more efficient method than manual coding. We explored the utility of (TLD, Kalal et al., 2010) to extract movement features from videos (e.g., gaze dynamics, whole body, leg or arm movements of both the parent and the infant). We showed that TLD tracked the selected features from these videos with high efficiency. Furthermore, we demonstrated that TLD in combination with CRQA can successfully extract interactive movement dynamics and enables efficient analysis of movement in videos. This is, to our knowledge, the first study to employ TLD in combination with CRQA to analyse PCI data.

### Application of TLD to face-to-face interactions

TLD succeeded in tracking the infant direction of looking and the toy that the parent was manipulating (accuracy > 90%). TLD tracked the infant direction of looking with higher accuracy than the object, which can be explained by more restricted movement of infants in relation to the moving object. Like many available trackers, the TLD algorithm did not report any value or produced false ones, when some data was missing. One of the main reasons for this is due to clutter, i.e., when the object of interest is difficult to discriminate from other objects in the scene (Maggio and Cavallaro, 2011). For instance, if the parent manipulates an object with similar color than his/her clothes or the background, the tracker can falsely place the object in those areas even if the object has moved away. Therefore, it is important to visually control the performance of TLD so the tracker does not produce false positives. Although this imposes a manual intervention on the side of the experimenter, this is certainly not as time consuming and does not introduce any observer bias as it is likely in the case of frame-by-frame manual coding. Other common problems in trackers are those related to occlusions or variations in object appearance (Ross et al., 2007). However, TLD is continuously updating the tracker and training the detection, learning in each frame new positions of the object, allowing also for its re-detection, even after a temporary occlusion. Therefore, we believe that TLD and trackers using similar based theory (e.g., Nebehay and Pflugfelder, 2013) can considerably facilitate movement analysis in pre-recorded videos.

To our knowledge, the movement categorization applied in this paper has not been used in the study of PCI before. Traditionally, movement coding schemes are more general and mostly qualitative (Chorney et al., 2014). This is probably due to the fact that a fine-grained categorization of movements as the one presented here would probably be time-consuming and costly. In fact, it would require performing a frame-by-frame analysis, categorizing the direction of movement of each feature of interest. Apart from being laborious, it would most likely be highly erroneous and unreliable between coders, since often the movement is so small that a human coder would have difficulties noticing it. We overcame these issues by applying TLD together with the automatic classification of movement. Such a computational approach allowed fast frame-by-frame movement extraction and efficient categorization of every movement, thus reducing potential errors.

Accurate quantification of movement during human interactions can also be carried out with instruments such as sensors and with complex and expensive camera systems. However, the use of automatic algorithms such as TLD may enhance the ecological validity of research on interactions. Apart from TLD there are other algorithms for movement quantification: Motion Energy Analysis (e.g., Ramseyer and Tschacher) or the frame-differentiating method (Paxton and Dale, 2012). However, TLD is a tracker, so its advantage in comparison with other ecological approaches is that it determines the direction of movement of a feature instead of quantifying how much movement there is in a pre-defined area of a video image. It is particularly useful in combination with the analysis that quantifies synchronization of partners during interactions, such as CRQA and it could prove useful in all analyses of large corpuses of naturalistic video recordings, e.g., from clinical assessments, for which there is no coding scheme available.

### Integration of TLD and CRQA

CRQA analysis of the time series extracted using TLD revealed the dynamics of coupling between the infant head turning and the toy animated by the parent. The coupling was not related to the behavioral task itself and it could not be attributed to random looking by the infant. We confirmed this by computing the recurrence, first, between the parent and shuffled version of the infant time series and second, between the parent and five randomly selected infants (Richardson and Dale, 2005; Dale et al., 2011a). The effect of movement coupling in mother-infant dyads as captured by cross recurrence profiles is different from recurrence due to task setting (as indicated by the random-paired profiles) as well as different from a random baseline obtained by shuffling the behavioral time series (see Figures 3, 4). We note that the overall mean recurrence of the random-paired baseline differed from the shuffled baseline. Although no coupling was found, the higher mean recurrence observed in the random-paired baseline could be attributed to some residual task-specific recurrence, which is higher when fewer movement categories are present.

To better quantify movement patterns, it is important to select an appropriate movement categorization. In this paper, simple and detailed coordinate systems were applied to the data. Although the recurrence profiles were similar in the toy and spinning tasks, they differed slightly in the feeding task (see Supplementary Materials for the results of the detailed coordinate system for the spinning toy and feeding tasks). This arises probably because the simple coordinate system by default collapses levels of movement detail into fewer bins, which might lead to information loss. In fact, the central recurrence peak in the simple coordinate system splits into two peaks when applying the detailed one. These two peaks probably describe two different movement patterns, which cannot be depicted with a simpler coordinate system. Thus, the selection of the coordinate system is critical for a correct understanding of the recurrent movement patterns.

Additionally, to interpret the CRQA lag profiles it is important to consider the position of interaction partners relative to the camera. For example, in the free play situation the interaction was happening mainly in the line of sight of the camera so both movements follow the same category, i.e., if the object moved to the left, the head of the infant also moved left when following it (Figure 1). In the spinning toy task, however, the interaction is happening perpendicular to the line of incidence of the camera, therefore having a mirrored movement. If both movements are categorized in the same way, the interpretation of the coordination will be more complex. The reason for this complex profile can be seen in Figures 8A–C. First, the mother leans forward and shows the functioning of the spinning toy to the infant (Figure 8A), which then leans forward to play with the object (Figure 8B) while the mother leans back (Figure 8C). This sequence is repeated throughout the interaction. Figure 8 illustrates how, for example, the mother and the infant, when approaching the spinning toy, fall into different movement categories (i.e., mother moved left and the infant–right). Therefore, those movements will not recur and a minimum will be found at the moment of highest coordination, when they approach the toy. The presence of movements in opposite directions is not a limitation itself, but it is an essential feature to consider together with the type of movement categorization and position of the camera, so that the CRQA lag profile can be correctly interpreted. Importantly, all these contingencies in behavior may be captured by integrating TLD and CRQA together.

Apart from revealing the coupling between behaviors of each partner, the CRQA may indicate who is leading and who is following during the interaction (e.g., Shockley et al., 2003; Dale et al., 2011a; Leonardi et al., 2016). In the moving toy task the maximum recurrence between the infant and the parent movements was found at −240 ms. Thus, the parent moved the object and the infant followed after about 240 ms. This coupling is supported by previous research, which showed that already 2-month-olds visually track objects moving back and forth (Von Hofsten and Rosander, 1997), while 5-month-olds are able to follow moving objects using anticipatory head and eye movements (e.g., Jonsson and Von Hofsten, 2003).

We further explored the quality of the coupling of the mother and the infant movements using anisotropic Cross-Recurrence Quantification Analysis (aCRQA, Cox et al., 2016). We found an asymmetry in the parameters with the horizontal values significantly higher than the vertical ones. The higher values obtained in the horizontal axis suggest that the parent's movement constitutes larger structures (i.e., high horizontal laminarity), it is trapped in relatively long periods during the infant movement (i.e., high horizontal trapping time) and it stays continuously in a single matching behavior for a long period (i.e., high maximum horizontal line) (Cox et al., 2016). In other words, the mother's hand movement was more stable, showed less variability and kept the direction of movement for longer in comparison with the infant's head movement. There are two possible explanations of this asymmetry. First, it could be due to the fact that the arc of movement of the toy is longer compared to the infant head movement. Second, the contribution of head movements to visual tracking in infants increases from early age (Bertenthal and Von Hofsten, 1998). At 5 months, however, head tracking still does not dominate as it does at a later age (Daniel and Lee, 1990; Von Hofsten and Rosander, 1997). Thus, this jerky movement increases the asymmetry in the aCRQA parameters since the infant is still not able to efficiently and smoothly pursue the moving object. Further studies of this issue are necessary to confirm whether this asymmetry decreases with age as the one we observed during the feeding task where the infant was older and the aCRQA parameters suggested a more synchronized interaction.

### Limitations of the current approach

In most of free play locomotor studies, the primary camera used for behavioral coding might move to obtain a better view during the interaction (e.g., Adolph, 2008). Although TLD is still able to track individual features in moving camera conditions (Kalal et al., 2010), the categorization of movement applied here would be affected by the camera displacement therefore leading to wrong categorizations. A possible solution would require a static reference point always present in the video in order to estimate the camera displacement to correct the tracked features. Moreover, during interactions outside constrained lab settings, the environment is typically more cluttered, occlusions occur more often and variations of the tracked features arise more frequently, which could affect the efficiency of the tracker.

The approach presented here offers a tool to analyse a range of fine-grained individual movements of parts of the body (e.g., head or limb movements). However, the age of the infants tested simplified the analysis since movement at 5.5 months of age is limited and as infants grow older, their movement patterns become more complex (Adolph and Franchak, 2017). However, limited locomotion is not a prerequisite for using TLD and large-scale movements of the head and the body can still be tracked at a later age; for instance, when mothers try to engage infants in mutual collaboration tasks. But as movements become more complex, TLD may become less efficient in tracking some specific, fine movements. For instance, TLD efficiently tracks hand movements but it would be more difficult to classify more specific hand movements such as grasping or finger movements using the present categorization. Likewise, as in Messinger et al. (2009), TLD would be able to track face movements or even particular face features (e.g., mouth, eyes) but it would fail to quantify a higher level of detail such as a smiling activity. Therefore, in some cases manual coding may still prove necessary to capture selected aspects of PCI. However, recent approaches are aiming at applying machine and deep learning techniques to quantify hand movements (Liu et al., 2014) or to detect facial expressions (Messinger et al., 2009). Combining these machine learning techniques with the approach presented here could prove fruitful.

In the case of infants moving in the camera's view, TLD allows to center the camera position in relation to the tracked feature. In this way, the feature of interest will be always centered in the video image, which may facilitate the application of machine learning algorithms to measure some movements. As a result, TLD can be used to quantify a movement (e.g., head movement) and center the camera position relative to it, and machine learning approaches can be used to extract more specific movements (e.g., smiling).

Data interpolation in TLD requires further consideration. In our case, in those episodes where missing values were reported by the tracker, a linear interpolation was assumed between the last known point and the first available. Although this proved sufficient here, where most of the movement mainly follows a linear path from left to right, a more complex categorization of the movement might need different assumptions in order to improve the recurrence between the movement of the parent and the infant during CRQA. For instance, infants lying on the floor have a restricted head movement from left to right with <180° rotation in comparison with the manipulated object, which moves more freely. When using a more fine-grained categorization, if an object moves in an up-right direction, the head movement of the infant due to the rotation of the head will most certainly fall into a down-left category, therefore not producing any recurrence in the analysis. In this case, a linear interpolation of the data will probably categorize the movement incorrectly. Thus, future studies need to further investigate the interpolation of missing data to the selected categorization of movement in order to correctly estimate the number of recurrences between movements.

Finally, our analysis was constrained to a restrictive interaction context in which parents animated an object and the infants followed them with their head and gaze. Thus, our approach may have a limited set of applications, given that face-to-face interactions are relatively infrequent in naturalistic studies (e.g., Deák et al., 2014). However, despite being rare, we believe that the study of face-to-face interactions is still important since the duration of face-to-face episodes during early interactions may have long-term effects on cognitive skills such as attention control (Niedzwiecka et al., 2017).

### Future directions

The approach to movement extraction and dynamical analysis outlined in this paper can be applied in multiple areas of developmental research. One possible application of this approach would be the analysis of parent-child locomotor behaviors, i.e., the approach and avoidance behaviors or changes in parent-child proximity distance (e.g., Peery and Crane, 1980). These variables are crucial in studies stemming from the attachment-related behaviors (Ainsworth, 1979). Under stable camera conditions this method could be used to track and categorize whole-body movements. Consequently, distances between the partners might be computed using the tracked features or even synchronization between the categorized movements could be computed using CRQA (e.g., Shockley et al., 2003).

A second potential line of work concerns the analysis of general movements of infants with developmental disabilities, such as cerebral palsy (e.g., Dimitrijević et al., 2016). Cerebral palsy in young infants is investigated by looking at their general movements and it is normally characterized by abnormal movements of reduced complexity, variability and fluency (e.g., Ferrari et al., 1990). Another potential application would be the study of infants at risk of autism. Several studies have reported that motor atypicalities are associated with later ASD and are a common comorbidity in people with autism (e.g., Teitelbaum et al., 1998; Zwaigenbaum et al., 2005). This approach might be a useful tool to quantify and analyse movement in these studies in search for intraindividual and interpersonal motor coordination disabilities.

## Conclusion

Our study sought to investigate the use of TLD to extract movement of each partner from video recordings of interactions during free-play of 5.5 months-old infants and their mothers. We demonstrated that automatic movement extraction from video recordings may considerably improve the efficiency of movement analysis in dyadic interactions. CRQA of extracted movement time series was proven to be a useful tool to examine the dyadic behavior as a system, allowing us to characterize the coordination between the movement of each interaction partner at different points in time and to determine leading patterns of behavior. Moreover, anisotropic CRQA showed that the differences in the symmetry of the parameters of recurrence plots may shed light on the quality of the movement that likely reflects individual differences in motor development.

## Author contributions

DL conceived the structure of the manuscript and performed the data analysis, GL helped with the statistical analysis and all authors have contributed writing the manuscript.

### Conflict of interest statement

The authors declare that the research was conducted in the absence of any commercial or financial relationships that could be construed as a potential conflict of interest.
