# Supplementary material for: Combining Recurrence Analysis and Automatic Movement Extraction from Video Recordings to Study Behavioral Coupling in Face-to-Face Parent-Child Interactions
Source: Front Psychol. 2017 Dec 19;8:2228. doi: 10.3389/fpsyg.2017.02228 (PMC5742271; doi:10.3389/fpsyg.2017.02228)
Supplement: Supplementary file 1 [file DataSheet1.pdf]

Supplementary Material

Combining Recurrence Analysis and Automatic Movement Extraction from Video Recordings to Study Behavioural Coupling in Face-to-Face Parent-Child Interactions

David, López Pérez, Giuseppe, Leonardi, Alicja, Niedźwiecka, Alicja, Radkowska, Joanna, Rączaszek-Leonardi and Przemysław, Tomalski

*** Correspondence:**

David López Pérez, david.lopez@psych.uw.edu.pl, Faculty of Psychology, University of Warsaw, 5/7, 00-183 Warsaw, Poland, tel. 0048 225549789

Przemysław Tomalski, p.tomalski@uw.edu.pl, Faculty of Psychology, University of Warsaw, 5/7, 00-183 Warsaw, Poland, tel. 0048 225549753

1 Supplementary Data

This supplementary file provides a step by step guide for the approach described in the paper. It details the procedure using a full face-to-face interaction example. Some of the Matlab functions that were used in the analysis are also provided together with this tutorial.

1.1 Tracking-Learning-Detection (TLD)

In this paper, version 1.0 of the TLD software was used to analyse the data, since newer versions have not been released in Matlab. The code and installation instructions can be found in https://github.com/zk00006/OpenTLD.

Once installed and compiled, the software contains a file called *run_TLD.m* that provides a working example of the TLD methodology. This example works on video acquired in real time. In order to make it work with pre-recorded videos, two steps are needed:

- The pre-recorded video has to be separated in individual frames.
- The code line establishing the input source needs to be changed as follows:

```
opt.source = struct('camera',0,'input',strcat(folder,'/'),'bb0',[]);
```

where *folder* points to the location of your video frames and *'camera', 0,* indicates to the TLD software not to look for the live camera but for a pre-recorded video. Once done, the *run_TLD.m* script can be run as it is.

The first thing the algorithm will do is ask for the object to be tracked. Thus, it will show the first frame of the video in which a box defining the object needs to be drawn. Figure 1 shows two examples. For instance, if we are interested in the object the mother is moving, a box can be drawn to contain the object or the hand the mother is using to animate the object. Alternatively, if we are interested in the infant's head movements, we can draw the box around the infant's face. It is important that the object we want to track has good contrast in comparison to the background image.

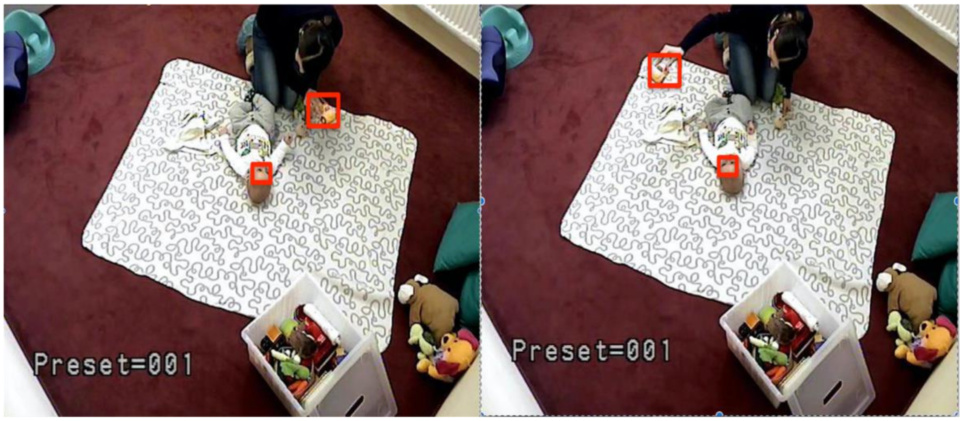

Figure 1. Example of how TLD was used to track the infant's focus of attention and the object that the mother held in front of the infant. The red bounding boxes represent these tracked features. Parents gave written consent to use the images in the publication.

Once the area is defined, the algorithm starts when the user double-clicks within the defined box. The algorithm will track the defined object frame by frame. The defined box is not stiff and it adapts in size to variations of size in the tracked object. There are two videos available with this tutorial showing how the algorithm tracks the defined object (https://mega.nz/#F!BjxnlCwK!IJWTQCn1BV20auBA0u3rQg). *Infant.mov* shows how the infant's head movements were tracked, while *Mother.mov* shows the tracking of the mother's hand movements.

When the algorithm finishes processing all the frames, the information about the tracked object will be saved in the output folder. In *run_TLD.m* this is determined by the opt.output field. Figure 2 shows an example of what the output of TLD looks like. The output file has five columns. The first four columns represent the coordinates of the bounding box relative to the video and the last column provides the information about the overall similarity of the pixels in the box is the content of the box in the current frame relative to the initially defined box. If the tracker loses temporarily the information about the object, the algorithm returns *NaN* values in all the columns.

```
327,190,351,214,1
326.86,189.77,351.25,214.11,0.98812
326.88,189.79,351.42,214.24,0.97109
326.8,189.79,351.54,214.36,0.983
326.64,189.85,351.9,214.87,0.92326
326.57,189.91,352.02,215.1,0.95817
326.61,189.97,352.06,215.14,0.97077
326.65,190.04,352.26,215.32,0.96929
326.8,190.03,352.44,215.32,0.94874
327.14,189.56,353.3,215.29,0.92858
327.27,189.64,353.51,215.44,0.90407
327.45,189.73,353.58,215.42,0.90053
327.53,190.03,353.46,215.55,0.8904
327.24,190.25,353.12,215.74,0.84661
326.92,190.32,352.86,215.87,0.81133
326.7,190.29,352.59,215.81,0.81334
326.74,190.4,352.58,215.85,0.74279
326.74,190.4,352.79,216.07,0.77424
326.7,190.47,352.64,216,0.68176
326.96,190.49,352.99,216.1,0.69122
327.18,190.56,353.02,216.02,0.67405
327.31,190.51,353.11,215.93,0.65454
327.33,190.56,353.03,215.89,0.62113
327.7,190.55,353.18,215.68,0.63524
```

Figure 2. Example of the output that TLD provides. It contains five columns. The first four columns represent the coordinates of the bounding box. The last field is the similarity between the object within the box and the object originally defined.

The output of TLD needs to be processed to create time series that can be used in subsequent Recurrence Quantification Analysis (Figure 3). Therefore, first, each set of coordinates is translated into the central coordinates of the tracked feature (Figure 3a). Thus, we use the information provided by the first four columns of the output file. Columns 1 and 3 contain the information about the x axis, while 2 and 4 about the y axis. The central coordinates of the box can then be computed using the average value for the x axis and the y axis, respectively. Then, these coordinates are compared with the central coordinates of the next frame to determine the direction of movement (Figure 3b). Finally, this movement is classified following two tentative systems of coordinates (Figure 3c). The function *transformCoordinates.m* automatizes the classification process. The function is defined as follows:

```
function coordinatesArray = transformCoordinates(xArray,yArray,Direction)
```

This function requires two input arrays, one identifying the central x-coordinates (xArray) and the other containing the central y-coordinates (yArray). Additionally, it needs to specify the number of directions of movement considered. Direction = 1 will calculate the detailed coordinate system with 9 possible directions of movement, while; Direction = 2 or 3 will calculate the simple coordinate system with 3 possible directions of movement. The difference between Direction equal to 2 and to 3 is that the left and right categorisation is inverted (see Figure 3c). This can be used to understand better any existing coordination between mirror behaviours (e.g. when two persons are facing each other perpendicular to the line of sight of the camera).

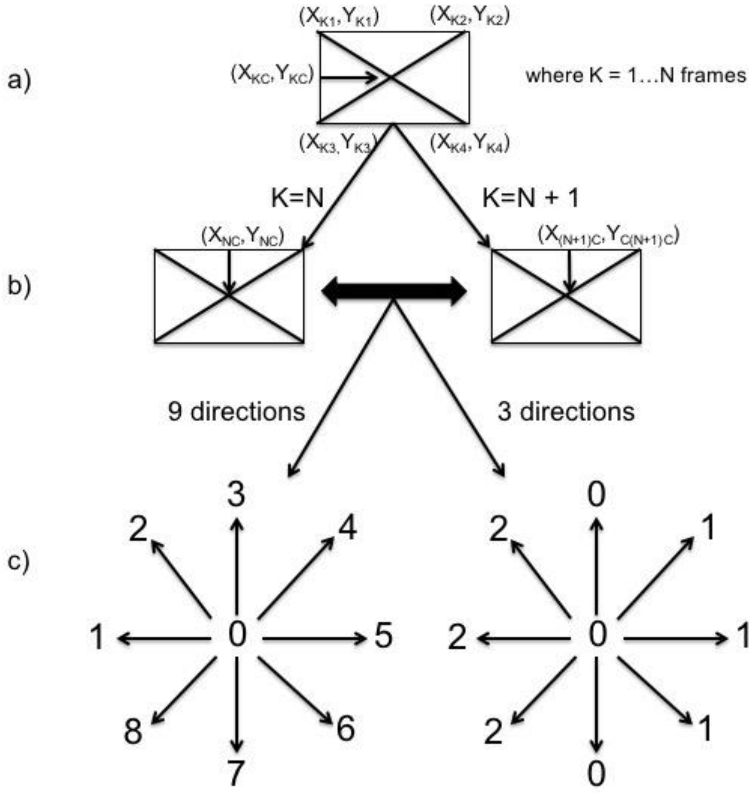

Figure 3. Categorisation of movement. The central coordinates of the TLD window are extracted (a) and compared to the next window central coordinates (b). The movement was classified following two systems of coordinates containing 9 and 3 categories of movement respectively (c).

The output of *transformCoordinates.m* is an array containing the categories of movement depending on the system of coordinates that has been selected. Figure 4 shows sample output of the simple (4a) and detailed coordinate systems (4b).

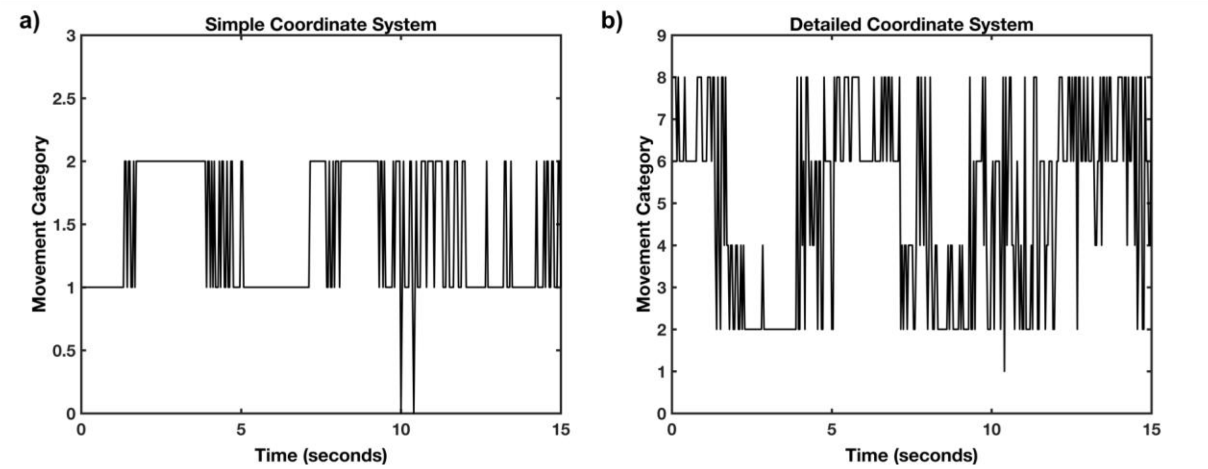

Figure 4. Sample classification of the mother's movement using the simple (4a) and detailed (4b) coordinate systems.

Cross-Recurrence-Quantification Analysis (CRQA)

Now, these transformed time series are ready for subsequent CRQA analysis. The analysis of recurrence rate near the main diagonal line of the recurrence plot allows to reconstruct a lag profile, which contains information about the coordination of those time series (Richardson & Dale, 2005). To construct the lab profiles, we used a translated version of the *drpdfromts* function present in the CRQA R-package (Coco & Dale, 2014). The function *drpdfromtsCat.m* provided along with this guide is defined as follows:

```
function [profile,maxrec,maxlag] = drpdfromtsCat(t1,t2,ws)
```

where *t1* and *t2* are two time series containing the categorised movement (e.g. the infant's head and mother's hand movements) and *ws* determines the size of the lag profile. For example, if we have a video recorded at 25 Hz and we want to generate 4-second lag profiles, our *ws* has to be equal to 100. *drpdfromtsCat.m* returns the computed lag profile (*profile*). If needed, it also returns the maximum value of recurrence of the lag profile (*maxrec)* as well as the position of this maximum (*maxlag*). Figure 5 shows the lag profiles for both systems of coordinates. These lag profiles correspond to the coordination of movements between the mother and the infant extracted from *Infant.mov* and *Mother.mov*:

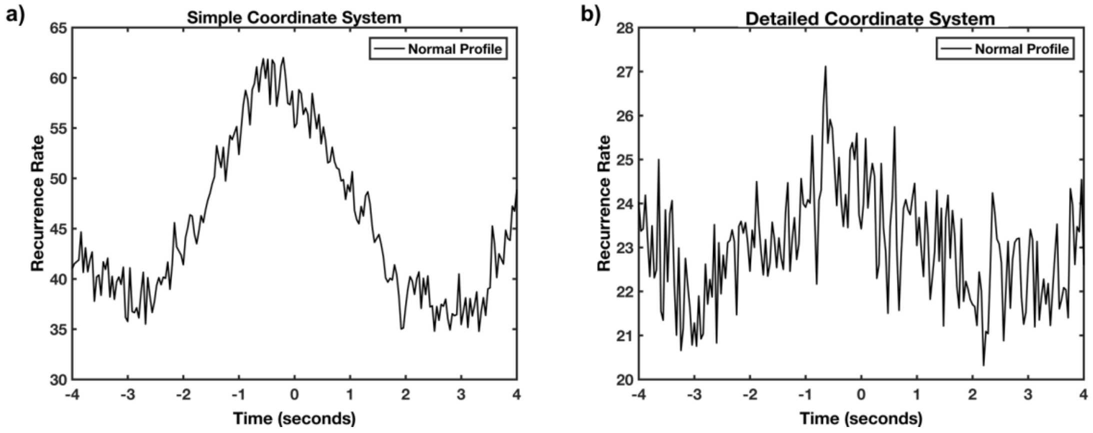

Figure 5. Example of the object-infant's focus lag profiles computed using diagonal-wise CRQA using the simple (5a) and the detailed (5b) coordinate systems.

The content of *t1* and *t2* is going to affect the interpretation of the lag profiles. For instance, if *t1* contains the mother's movement and *t2* the infant's movement information, the left part of the profile relates to mother leading actions and the right part to infant leading actions. Therefore, if the recurrence maxima are located on the left side, it means that the mother is leading the action. If the maxima are located at 0 seconds, it suggests perfect coordination. Finally, if they are located on the right side, it means that the infant is leading the action. The crucial point is to always be consistent in the order given to the *drpdfromtsCat.m* function. In other words, *t1* and *t2* must always contain the same type of behaviour (e.g. the mother's movement or the infant's head movement) so results are consistent when averaging the profiles.

Normalisation of the lag profiles

In order to prove that the coordination patterns are real, we need to first check that the profile didn't arise by chance. This step consists of calculating the lag profile with one or both time series shuffled to see if the shape of the profile changes. The random shuffling of a time series can be done using the following command in Matlab:

```
infantMovementShuffled = infantMovement(randperm(numel(infantMovement)));
```

After the shuffling is complete, the lag profile needs to be calculated again using the *drpdfromtsCat.m* function. Figures 6a and 6b show the comparison of the profiles from Figure 5 with their shuffled versions. During the shuffled baseline calculation, it is advisable to run the randomisation of the time series several times (e.g. 100 times bootstrapping) and obtain an average shuffled baseline.

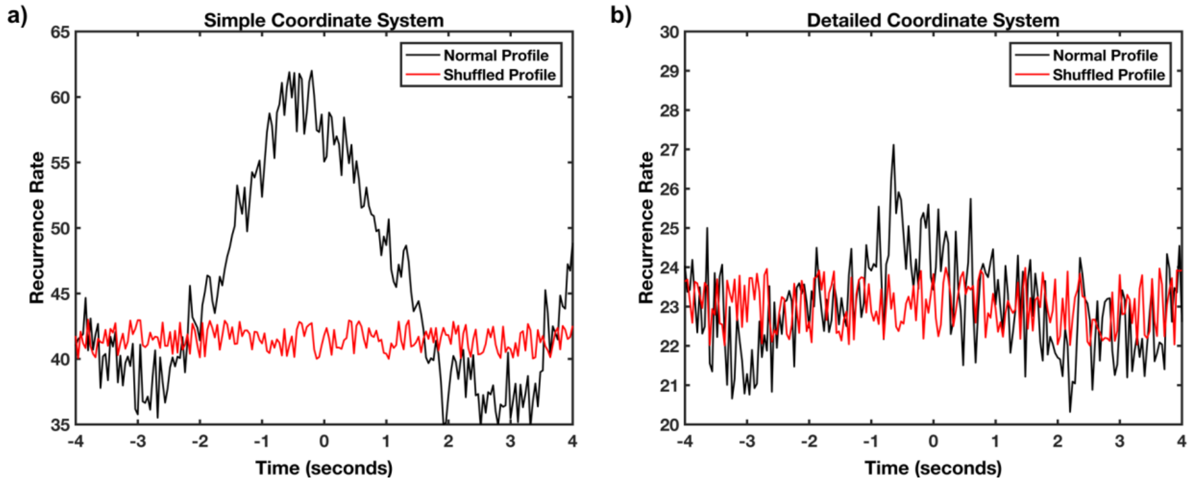

Figure 6. Example of the object-infant's focus of attention (black) and object-shuffled infant's focus of attention (red) lag profiles computed using diagonal-wise CRQA using the simple (6a) and the detailed (6b) coordinate systems. Only mean values are showed for the random-paired profiles.

Likewise, we need to test that the profile is not task-specific. This recurrence refers to the baseline of recurrence between infants who share the same free-play task but show different behaviour (Richardson & Dale, 2005). In the current example, the mother's hand movement was random-paired with the head movements of 5 infants from different dyads. During random-pairing the time series may possess different length. In this case, the last part of the longest time series might get trimmed to match the length of the shortest one. Figure 7a and 7b show the comparison of the profiles from Figure 5 with their random-paired versions.

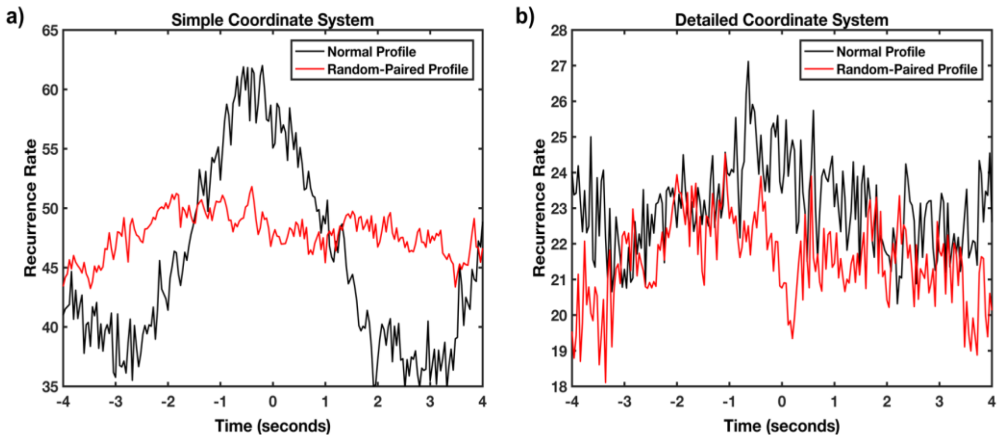

Figure 7. Example of the object-infant's focus of attention (black) and object-randomised infant's focus of attention (red) lag profiles computed using diagonal-wise CRQA for the simple (7a) and the detailed (7b) coordinate systems. Only mean values are showed for the random-paired profiles.

In general, if the coordination is real, both the shuffled and random-paired baselines should be relatively flat and significantly below the peak of coordination. Statistical comparison between profiles can be done applying Linear Mixed Models in R (R Core Team, 2016) using packages lme4 (Bates, Mächler, Bolker & Walker, 2015) and lmerTest (Kuznetsova, Brockhoff & Christensen, 2016), which allow to check the statistical significance for the estimated coefficients of the model using t-tests and the Satterthwaite approximations for the degrees of freedom.

Asymmetric CRQA

The analysis of lag profiles is a useful method to find coordination between two time series. However, RQA provides a wider range of measures that help find out more about the dynamics of two systems. The nature of categorical time series impacts the recurrence plots in such a way that most of the recurrences are arranged to form rectangular or vertical line structures (see Figure 8 for example).

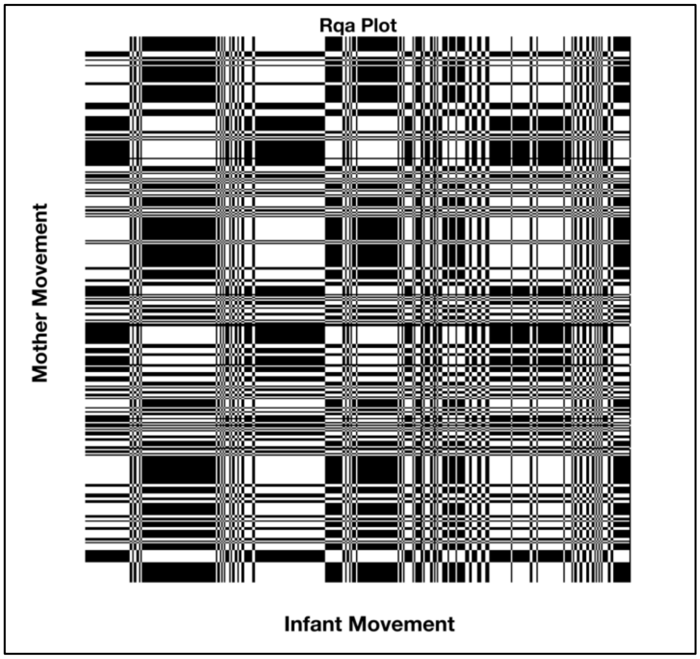

Figure 8. Example of the RQA plot of the object-infant's focus of attention using the simple coordinate system.

Most of the traditional RQA measures focused on quantifying diagonal structures (Webber & Zbilut, 1994). However, recently, a new technique, called Anisotropic CRQA (aCRQA), has been proposed to overcome this problem (Cox et al., 2016). aCRQA can provide information about the coupling between two time series by quantifying the vertical and the horizontal structures in the plot. To calculate these values, we used the *crqa.m* function provided as part of the CRP Toolbox for Matlab (Marwan, Wessel, Meyerfeldt, Schirdewan & Kurths, 2002; Marwan & Kurths, 2002). The Matlab code can be downloaded from http://tocsy.pik-potsdam.de/CRPtoolbox/. Both the vertical and the horizontal values can be calculated using the following commands:

```
verticalValues = crqa(RQAData(:,1), RQAData(:,2),M,T,E,[],normMethod,normalisation,'sil');

horizontalValues = crqa(RQAData(:,2), RQAData(:,1),M,T,E,[],normMethod,normalisation,'sil');
```

crqa.m returns a large number of RQA parameters (see *help crqa* for more details) but in order to quantify the asymmetric parameters, we need to focus only on three of them. First, the

trapping time (TT), which indexes the average time spent by the participants in the various movement categories mapped in the time series:

rqaResults.vTT = verticalValues(:,7);

Second, the laminarity (LAM), which indexes the general level of persistence in some particular state of one of the time series:

rqaResults.vLAM = verticalValues(:,6);

Finally, the maximum line (MaxL), which gives the longest time spent by interaction partners in a single state:

rqaResults.vMaxL = verticalValues(:,8);

The same can be done for the horizontal lines:

rqaResults.hTT = horizontalValues(:,7);

rqaResults.hLAM = horizontalValues(:,6);

rqaResults.hMAxL = horizontalValues(:,8);

Any asymmetry found between vertical and horizontal lines would reveal an asymmetric dynamic attunement between the tracked movements. For example, if the movements of the mother and infant recur and have the same duration in time a perfect symmetry would be found. However, such coordination during interactions is difficult to achieve and the asymmetry between movements starts to arise.

We have seen that the *crqa.m* function requires a large number of input parameters. For categorical time series, these values are typically as follows:

- *RQAData*(:,1) and *RQAData*(:,2), i.e., the two input time series containing the categorised movement. The reason why the *crqa.m* function needs to be run twice is that it only returns information about the vertical lines. Therefore, it is necessary to invert the time series input in order to invert the RQA plot; and
- *M, which* is the embedded dimension. For categorical time series as the one presented here, this value should be left at 1.
- *T*, that is, the delay. It has been suggested that the delay value does not have a strong impact on the RQA results. A default value of 1 is normally used.
- *E, which* is the radius. Since in categorical data we are interested in recurring only those categories which have equal values, the radius size has to be kept smaller than differences between categories. For example, if we do not want category equal to 2 to recur with category equal to 3, then the radius has to be set to a value smaller than 3 minus 2 (i.e., smaller than 1).
- *normMethod*, which constitutes the type of method used to find neighbours in the distance space. Since categorical data does not require normalisation, this value can have any of the possible normalisation values that *crqa.m* accepts.
- *Normalisation*, which indicates the data that needs to be normalised using the selected *normMethod*. Categorical data does not require normalisation, thus, this value is set to *'non'*.
- *'sil'*, which silences any output dialog that the function creates.

Summary

Figure 9 summarises the methodology presented in this guide. First, *runTLD.m* is executed to track the features of interest (Figure 9a). Second, the output of TLD for each tracked feature is categorised for two different systems of coordinates (i.e. simple and detailed coordinate system) using *transformCoordinates.m* (Figure 9b). Subsequently, the *drpdfromtsCat.m* function is used to compute the lag profiles in search for any sign of coordination between the movement-categorised time series (Figure 9c). Finally, the asymmetric dynamics of the time series are determined using twice the *crqa.m* function to quantify the vertical and horizontal structures of the RQA plot (Figure 9d).

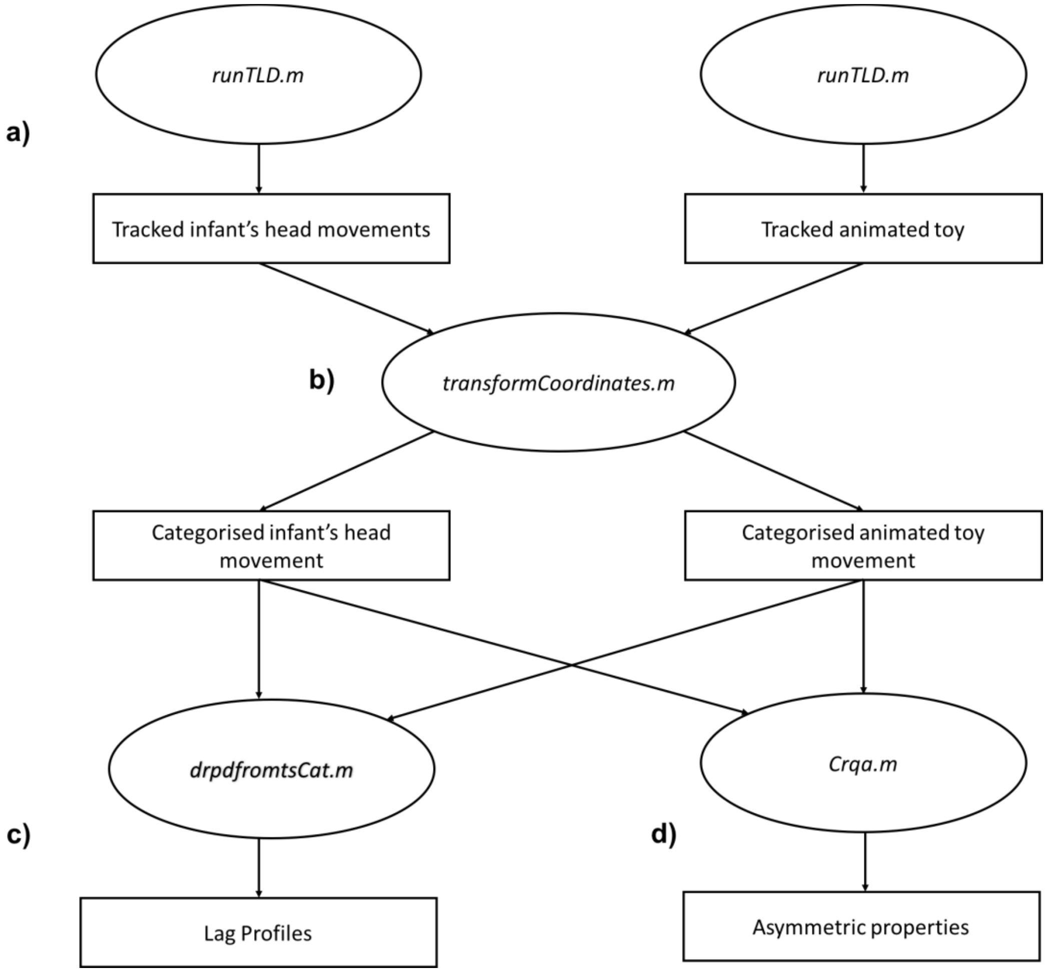

Figure 9 Summary of the methodology presented in this guide. First, runTLD.m is executed to track the features of interest (9a). Second, the output of TLD for each tracked feature is categorised using transformCoordinates.m (9b). Subsequently, the drpdfromtsCat.m function is used to compute the lag profiles (9c). Finally, the asymmetric dynamics of the time series are determined using twice the crqa.m function(9d).

References

1. Bates, D., Mächler, M., Bolker, B., & Walker, S. (2015). Fitting Linear Mixed-Effects Models Using lme4. *Journal of Statistical Software, 67*(1), 1–48. doi:10.18637/jss.v067.i01
2. Coco, M. I., & Dale, R. (2014). Cross-recurrence quantification analysis of categorical and continuous time series: An R package. Frontiers in Psychology, 5. doi:10.3389/fpsyg.2014.00510
3. Cox, R. F., Steen, S. V., Guevara, M., Jonge-Hoekstra, L. D., & Dijk, M. V. (2016). Chromatic and Anisotropic Cross-Recurrence Quantification Analysis of Interpersonal Behavior. In Springer Proceedings in Physics Recurrence Plots and Their Quantifications: Expanding Horizons, 209-225. doi:10.1007/978-3-319-29922-8_11
4. Kalat, Z., Mikolajczyk, K., and Matas, J. (2010). Tracking-Learning-Detection. Journal IEEE Transactions on Pattern Analysis and Machine Intelligence, 34 (7), 14091422. doi: 10.1109/TPAMI.2011.239.
5. Kuznetsova A., Brockhoff P.B. & Christensen R.H.B. (2016). lmerTest: Tests in Linear Mixed Effects Models. R package version 2.0-33. https://CRAN.R-project.org/package=lmerTest.
6. Marwan, N., Kurths, J. (2002) Nonlinear analysis of bivariate data with cross recurrence plots. *Physical Letters A, 302.*
7. Marwan, N., Wessel, N., Meyerfeldt, U., Schirdewan, A., & Kurths, J. (2002). Recurrence-plot-based measures of complexity and their application to heart-rate-variability data. *Physical Review E, 66*(2). doi:10.1103/physreve.66.026702
8. Richardson, D. C., & Dale, R. (2005). Looking To Understand: The Coupling Between Speakers' and Listeners' Eye Movements and Its Relationship to Discourse Comprehension. Cognitive Science, 29(6), 1045-1060. doi:10.1207/s15516709cog0000_29
9. Webber, C. L., Jr. & Zbilut, J. P. (1994). Dynamical assessment of physiological systems and states using recurrence plot strategies. Journal of Applied Physiology, 76, 965–973.
