# Supplementary material for: Combining Recurrence Analysis and Automatic Movement Extraction from Video Recordings to Study Behavioral Coupling in Face-to-Face Parent-Child Interactions
Source: Front Psychol. 2017 Dec 19;8:2228. doi: 10.3389/fpsyg.2017.02228 (PMC5742271; doi:10.3389/fpsyg.2017.02228)
Supplement: Supplementary file 2 [file DataSheet2.DOCX]

Supplementary Material

**Combining Recurrence Analysis and Automatic Movement Extraction from Video Recordings to Study Behavioural Coupling in Face-to-Face Parent-Child Interactions**

**David, López Pérez, Giuseppe, Leonardi, Alicja, Niedźwiecka, Alicja, Radkowska, Joanna, Rączaszek-Leonardi and Przemysław, Tomalski**

*** Correspondence:**

David López Pérez, [david.lopez@psych.uw.edu.pl](mailto:david.lopez@psych.uw.edu.pl), Faculty of Psychology, University of Warsaw, 5/7, 00-183 Warsaw, Poland, tel. 0048 225549789

Przemysław Tomalski, [p.tomalski@uw.edu.pl](mailto:p.tomalski@uw.edu.pl), Faculty of Psychology, University of Warsaw, 5/7, 00-183 Warsaw, Poland, tel. 0048 225549753

# Supplementary Figures and Tables

This supplementary material contains the results of the detailed coordinate system of the feeding and spinning toy tasks.

## Feeding Task

Bonferroni-adjusted t-tests showed significant differences between the original recurrence profile with the shuffled one in the four windows (t_1_(61) = 5.44, p_1_ < 0.001, d = 1.17; t_2_(61) = 6.25, p_2_ < 0.001, d = 1.40; t_3_(61) = 21.76, p_3_ < 0.001, d = 4.23; t_4_(61) = 29.02, p_4_ < 0.001, d = 5.37).


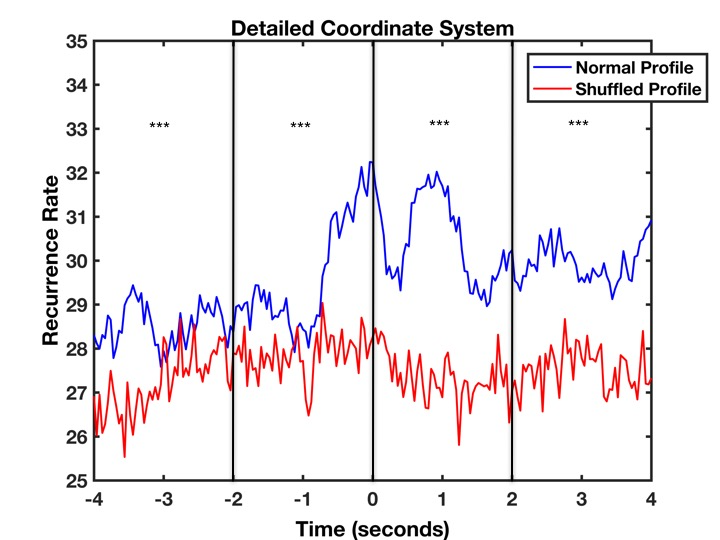


**Supplementary Figure 1.** *Lag profile between mother and infant body movements during feeding task computed using diagonal-wise CRQA for the simple coordinate system. The asterisks represent in which windows there were significant differences between the original recurrence profile with the shuffled one (p < 0.001(*** )).*

## Spinning-Toy Task

Bonferroni-adjusted t-tests showed significant differences between the original recurrence profile with the shuffled one in the four windows (t_1_(61) = -0.79, p_1_ = -0.14, d = -0.07; t_2_(61) = -2.44, p_2_ = 0.017, d = -0.47; t_3_(61) = -9.29, p_3_ < 0.001, d = -1.73; t_4_(61) = 1.99, p_4_ = 0.05, d = 0.39).


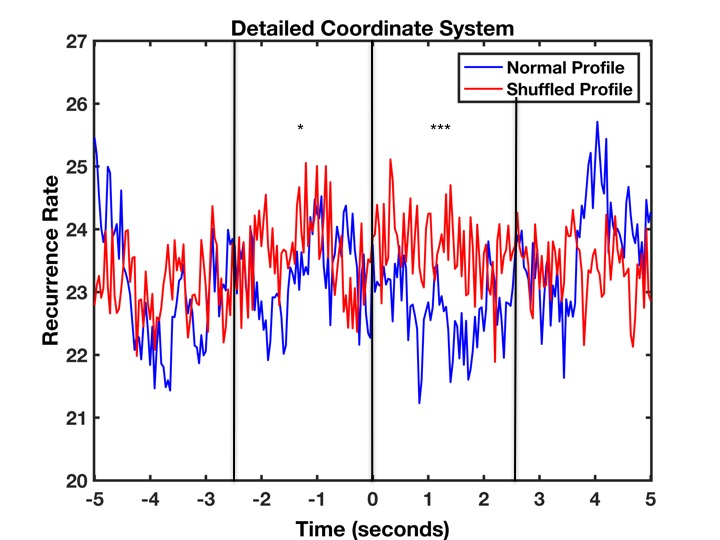


**Supplementary Figure 2.** *Lag profile between mother and infant body movements during spinning task computed using diagonal-wise CRQA for the simple coordinate system. The asterisks represent in which windows there were significant differences between the original recurrence profile with the shuffled one ( p<0.05 (*), p < 0.001(***)).*
